# The coordination chemistry of Cm^III^, Am^III^, and Ac^III^ in nitrate solutions: an actinide L_3_-edge EXAFS study†
†LA-UR-18-22688.
‡
‡Electronic supplementary information (ESI) available. See DOI: 10.1039/c8sc02270d


**DOI:** 10.1039/c8sc02270d

**Published:** 2018-08-01

**Authors:** Maryline G. Ferrier, Benjamin W. Stein, Sharon E. Bone, Samantha K. Cary, Alexander S. Ditter, Stosh A. Kozimor, Juan S. Lezama Pacheco, Veronika Mocko, Gerald T. Seidler

**Affiliations:** a Los Alamos National Laboratory, , Los Alamos , New Mexico 87545 , USA . Email: stosh@lanl.gov; b Department of Physics , University of Washington , Seattle , Washington 98195-1560 , USA; c Stanford University , Stanford , California 94305 , USA

## Abstract

Understanding actinide(iii) (An^III^ = Cm^III^, Am^III^, Ac^III^) solution-phase speciation is critical for controlling many actinide processing schemes, ranging from medical applications to reprocessing of spent nuclear fuel. Unfortunately, in comparison to most elements in the periodic table, An^III^ speciation is often poorly defined in complexing aqueous solutions and in organic media. This neglect – in large part – is a direct result of the radioactive properties of these elements, which make them difficult to handle and acquire. Herein, we surmounted some of the handling challenges associated with these exotic 5f-elements and characterized Cm^III^, Am^III^, and Ac^III^ using An^III^ L_3_-edge X-ray absorption spectroscopy (XAS) as a function of increasing nitric acid (HNO_3_) concentration. Our results revealed that actinide aquo ions, An(H_2_O)_*x*_^3+^ (*x* = 9.6 ± 0.7, 8.9 ± 0.8, and 10.0 ± 0.9 for Cm^III^, Am^III^, and Ac^III^), were the dominant species in dilute HNO_3_ (0.05 M). In concentrated HNO_3_ (16 M), shell-by-shell fitting of the extended X-ray fine structure (EXAFS) data showed the nitrate complexation increased, such that the average stoichiometries of Cm(NO_3_)_4.1±0.7_(H_2_O)_5.7±1.3_^(1.1±0.2)–^, Am(NO_3_)_3.4±0.7_(H_2_O)_5.4±0.5_^(0.4±0.1)–^, and Ac(NO_3_)_2.3±1.7_(H_2_O)_8.3±5.2_^(0.7±0.5)+^ were observed. Data obtained at the intermediate HNO_3_ concentration (4 M) were modeled as a linear combination of the 0.05 and 16 M spectra. For all three metals, the intermediate models showed larger contributions from the 0.05 M HNO_3_ spectra than from the 16 M HNO_3_ spectra. Additionally, these efforts enabled the Cm–NO_3_ and Ac–NO_3_ distances to be measured for the first time. Moreover, the An^III^ L_3_-edge EXAFS results, contribute to the growing body of knowledge associated with Cm^III^, Am^III^, and Ac^III^ coordination chemistry, in particular toward advancing understanding of An^III^ solution phase speciation.

## Abstract 2

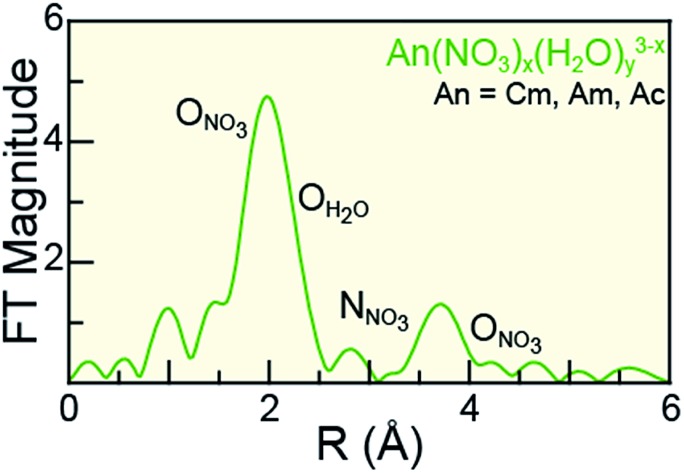
Cm^III,^ Am^III^, and Ac^III^ have been characterized by solution L_3_-edge X-ray absorption spectroscopy as a function of nitric acid concentration. This enabled the first experimental determination of Cm and Ac nitrate distances.

## Introduction

The actinide(iii) (An^III^) cations of Cm^III^, Am^III^, and Ac^III^ occupy central roles in numerous nuclear technologies important to society. These range from the medical applications in the targeted alpha therapeutic treatment of disease (Ac^III^)^1^–^3^ to being critical components in advanced nuclear fuel cycles (Am^III^ and Cm^III^).^4^–^9^ Solving technical problems in these areas require detailed understanding of fundamental +3 f-element chemistry. Unfortunately, aside from a limited number of experimental and computational studies,^10^–^21^ the chemistry of Cm^III^, Am^III^, and Ac^III^ is underdeveloped in comparison to the d-block, main group, and many other 4f- and 5f-elements. This discrepancy – in large part – is related to the rarity of these elements combined with handling difficulties that accompany the radioactive Cm, Am, and Ac isotopes.

This manuscript documents our latest effort to address needs for advancing fundamental Cm^III^, Am^III^, and Ac^III^ chemistry. We focused on characterizing the coordination chemistry of these elements in an aqueous environment that contained actinide complexation agents, namely within nitric acid (HNO_3_) solutions. These results are of particular relevance, given the importance of HNO_3_ matrices in An^III^ separation processing. For Cm^III^ and Am^III^, HNO_3_ solutions find widespread application in almost every advanced nuclear fuel processing flow chart.^4^,^22^–^24^ Additionally, HNO_3_ is widely used in the production of ^225^Ac for medical purposes (targeted alpha therapy), both in terms of purifying ^225^Ac from ^232^Th targets irradiated with high energy protons^25^–^27^ and when isolating ^225^Ac from ^229^Th generators.^28^–^30^ Towards these ends, we contribute an An^III^ L_3_-edge X-ray absorption spectroscopy (XAS) study focused on characterizing Cm^III^, Am^III^, and Ac^III^ solution-phase coordination chemistry as a function of increasing HNO_3_ concentration. Our data provided the first An^III^–NO_3_ bond distance measurements for Cm^III^ and Ac^III^ of any kind (*i.e.* solid or solution) and represented the first Am^III^–NO_3_ measurement made in HNO_3_ solutions. We observed that at low HNO_3_ concentrations (0.05 M), Cm^III^, Am^III^, and Ac^III^ existed as aquo ions. The propensity of NO_3_^–^ to complex the An^III^ cations increased with increasing HNO_3_ concentration, such that in HNO_3_ (16 M) solutions there were 2 to 4 bound NO_3_^–^ ligands. The results have been presented in the context of the limited number of HNO_3_ speciation studies reported previously.

## Results and discussion

### Sample preparation

Samples were generated by first preparing chemically pure Cm^III^, Am^III^ and Ac^III^ stock solutions, as previously described.^12^,^20^,^31^ Next, aliquots that contained Cm^III^ (0.5 mg in 0.5 mL, 4.0 mM), Am^III^ (0.5 mg in 0.5 mL, 4.1 mM), and Ac^III^ (28 μg in 0.3 mL, 0.4 mM) were heated to soft dryness. The resulting residues were dissolved in the desired concentration of nitric acid (HNO_3_; 0.05, 4, or 16 M). This process was repeated for a total of three times to ensure that the final HNO_3_ concentrations were as close to 0.05, 4 and 16 M as possible. Samples were then loaded into XAS holders equipped with three layers of containment to guard against release of radiological material during shipment and data acquisition. Next, the holders were shipped to the Stanford Synchrotron Radiation Lightsource (SSRL) for XAS analysis at the An^III^ L_3_-edge on beam line 11-2.

### An^III^ L_3_-edge X-ray absorption near edge spectroscopy (XANES)

**Fig. 1:**
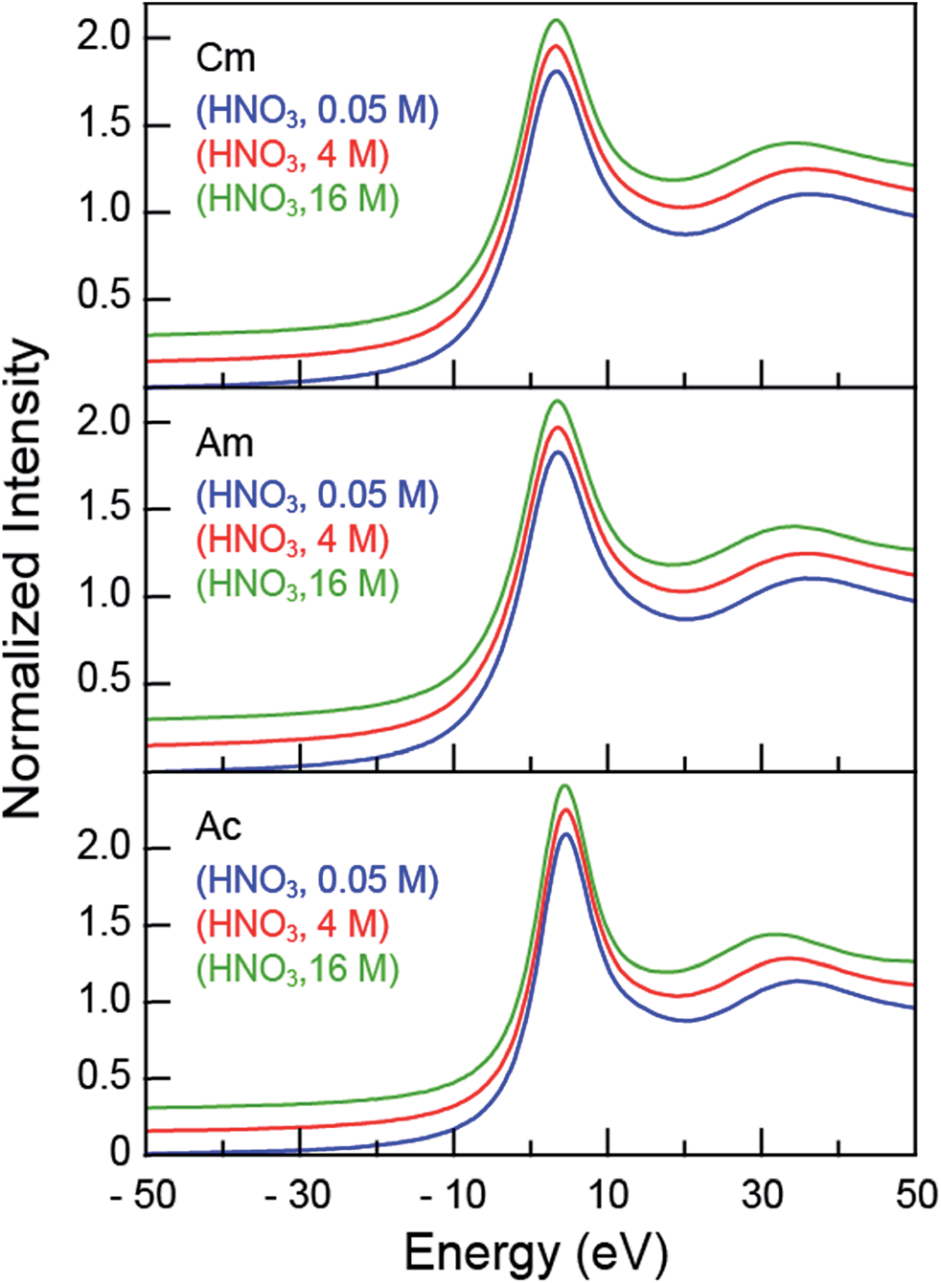
The background subtracted and normalized room temperature solution-phase An^III^ L_3_-edge XANES spectra of An^III^ (An = Cm^III^, top; Am^III^, middle; Ac^III^, bottom) cations dissolved in HNO_3_ (0.05 M, blue trace; 4 M, red trace; and 16 M, green trace). Spectra are displayed with a slight y-offset for clarity.

The room temperature Cm^III^, Am^III^ and Ac^III^ L_3_-edge XANES spectra obtained from aqueous solutions that contained increasing amounts of nitric acid (HNO_3_; 0.05, 4, 16 M) were background subtracted and normalized (Fig. 1). Each spectrum contained a pronounced absorption peak superimposed on an ionization threshold. From the perspective of the free ion, the edge-feature could be crudely described as originating from electric-dipole allowed transitions from the actinide 2p-orbitals to unoccupied states that contained actinide 6d-character, *i.e.* for Ac^III^ 2p^6^···5f^0^ 6d^0^ → 2p^5^···5f^0^ 6d^1^.^32^,^33^ The inflection points and peak maxima were determined graphically where the second derivatives (inflection point) and first derivatives (peak maxima) of the data equaled zero (Table 1). These values were impacted marginally by changes in HNO_3_ concentration: the inflection points for Cm^III^ were centered around 18 976 eV, for Am^III^ near 18 514 eV, and for Ac^III^ close to 15 875 eV. Based on our previous experience in reproducing actinide L_3_-edge features,^12^,^21^,^34^–^41^ uncertainties in edge and peak positions were estimated to be on the order of 0.2 eV. Hence, for a given element (Cm^III^, Am^III^, or Ac^III^), the 0.05, 4, and 16 M absorption edges and peak positions were nearly equivalent.

**Table 1:** Inflection points and peak positions (eV) of the room-temperature Cm^III^, Am^III^, and Ac^III^ L_3_-edge solution-phase XANES spectra of An^III^ dissolved in HNO_3_ (0.05, 4 and 16 M). The Cm^III^ and Am^III^ spectra were calibrated to the peak maximum of a Zr foil (18 013.3 eV) while the Ac^III^ spectra were calibrated to the first inflection point of a RbCl pellet (15 203.8 eV)

	Inflection point (eV)	Peak position (eV)	2^nd^ peak position (eV)
Cm aquo (1 M HClO_4_),^47^^*a*^	18 973.0	—	—
Cm (HNO_3_, 0.05 M)	18 976.4	18 980.3	19 013.5
Cm (HNO_3_, 4 M)	18 976.3	18 980.2	19 012.9
Cm (HNO_3_, 16 M)	18 976.3	18 980.2	19 011.4
Am (0.11 M HO_3_SCF_3_),^21^	18 514.3	18 517.9	
Am (HNO_3_, 0.05 M)	18 514.0	18 517.5	18 550.6
Am (HNO_3_, 4 M)	18 513.8	18 517.4	18 549.9
Am (HNO_3_, 16 M)	18 513.8	18 517.4	18 547.1
Ac (0.11 M HO_3_SCF_3_),^12^	15 874.3	15 876.9	
Ac (HNO_3_, 0.05 M)	15 874.7	15 877.6	15 908.1
Ac (HNO_3_, 4 M)	15 874.6	15 877.5	15 906.7
Ac (HNO_3_, 16 M)	15 874.6	15 877.5	15 905.0

^*a*^The Cm aquo complex in 1 M HClO_4_ was calibrated with Nb foil (18 986 eV).

Although the absorption peak position showed essentially no dependence on the HNO_3_ concentration, changing the HNO_3_ concentration from 0.05 M to 16 M had a marked impact on the energy of the post-edge feature approximately 30 to 40 eV above the inflection point (Fig. 1). This peak marks the first extended X-ray absorption fine structure (EXAFS) oscillation. Increasing the HNO_3_ concentration systematically lowered the energy for the oscillation maximum (Table 1). For Cm^III^, moving from 0.05 M to 4 M HNO_3_ caused a 0.6 eV oscillation maximum decrease. Similarly, moving from 4 M to 16 M HNO_3_ caused a –2.1 eV energy shift. Similar trends were observed for Am^III^ and Ac^III^, albeit the 3.5 (Am^III^) and 3.1 (Ac^III^) energy shifts were larger. Dependence of the post-edge line-shape on the HNO_3_ concentration foreshadowed structural changes that accompanied coordination of An^III^ cations by NO_3_^–^ ligands, which were revealed when the EXAFS spectra were rigorously analyzed.

### An^III^ L_3_-edge extended X-ray absorption fine structure (EXAFS) spectroscopy

**Fig. 2:**
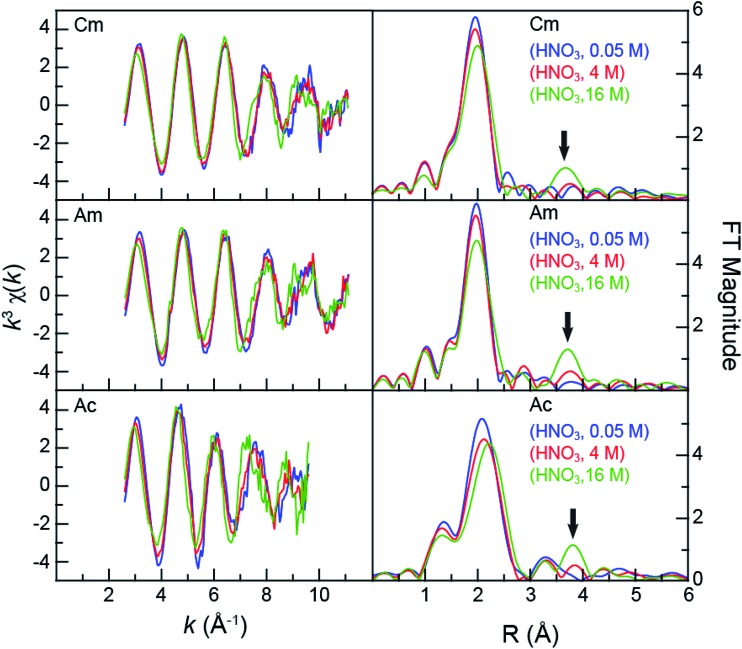
Left – the room temperature An^III^ L_3_-edge EXAFS function *k*^3^*χ*(*k*) from An^III^ (An = Cm^III^, top; Am^III^, middle; Ac^III^, bottom) cations dissolved in HNO_3_ (0.05 M, blue trace; 4 M, red trace; and 16 M, green trace). Right – the Fourier Transform of *k*^3^-EXAFS spectra. The black arrow emphasizes the growing nitrate contribution to the spectra.

Speciation metrics (*i.e.* coordination numbers and bond distances) for solution-phase samples were extracted from the *k*^3^*χ*(*k*) solution-phase measurements (Fig. 2). For Cm^III^ and Am^III^ – present in relatively high concentrations (0.5 mg in 0.5 mL per sample) – high quality data were obtained from 2.7 to 11 Å^–1^ (in *k-*space). This energy range provided shell-by-shell resolution in the Cm^III^ and Am^III^ measurements to be approximately 0.19 Å (resolution = π/2Δ*k*; in *R*-space). The Cm^III^ and Am^III^ L_3_-edge *k*^3^*χ*(*k*) spectra were quite similar and changed uniformly with increasing HNO_3_ concentration (Fig. 2). The first three oscillations were nearly superimposable despite the changes in HNO_3_ concentration. Upon reaching the fourth and fifth oscillations, some dependence on the HNO_3_ concentration became apparent. For example, moving from 0.05 M to 4 M HNO_3_ caused a subtle shoulder to emerge in the fifth oscillation (*ca.* 9 Å^–1^). Changing to concentrated HNO_3_ (16 M) increased the magnitude of this shoulder and caused a second shoulder to emerge on the low energy side of the fourth oscillation (*ca.* 7.5 Å^–1^). These line shape changes suggested that some of the scattering pathways were moving out of phase.

In general, the Ac^III^ spectra were similar to those of Cm^III^ and Am^III^. For example, increased HNO_3_ concentrations had little impact on the first three oscillations and showed evidence of out-of-phase scattering pathways for the fourth and fifth oscillations. The Ac^III^ L_3_-edge data differed in two notable ways. First, the signal-to-noise ratio was smaller, on account of the smaller quantity of Ac^III^ (28 μg). This restricted the energy range over which high quality data were available; from 2.7 to 9.5 in *k*-space (resolution = 0.23 Å in *R*-space). Second, the EXAFS oscillation frequency increased in comparison to those of Cm^III^ and Am^III^. This frequency increase was somewhat expected. For example, the frequency in *k-*space (left, Fig. 2) is inversely related to the interatomic distance. Higher frequencies result from longer bonds. As shown in Fig. 2, the large Ac^III^ ionic radius^42^ should provide longer bond distances (higher oscillation frequencies) than those for Cm^III^ and Am^III^, as long as the analytes have similar chemical compositions. Given the observed change in frequency upon moving from Cm^III^ and Am^III^ to Ac^III^ and the similar interference pattern, these data suggested – superficially – that the Ac^III^ speciation was similar to that of Am^III^ and Cm^III^.

Closer examination of the Cm^III^, Am^III^, and Ac^III^ L_3_-edge EXAFS spectra supported the proposition that chemical speciation was similar for these three compounds, revealing only subtle differences in Cm^III^, Am^III^, and Ac^III^ coordination environments. The experimental data were analyzed using well-established shell-by-shell curve fitting techniques.^43^ Interpretations of the data were guided by identifying scattering pathways using FEFF8 (ref. 44 and 45) and DFT geometry optimized An^III^ structures that contained a combination of water molecules and bidentate nitrate ligands, An(H_2_O)_9–2*x*_(NO_3_)_*x*_^3–*x*^ (An = Cm^III^, Am^III^, Ac^III^; *x* = 0, 1, 2, 3). The coordination numbers (CN), bond lengths (*R*), Debye–Waller factors (*σ*^2^), and energy shifts (*E*_0_) were allowed to converge to reasonable values. The amplitude reduction factor was set to 0.9. The fitting results have been summarized and compared with other relevant EXAFS studies in Table 2.^12^,^21^,^46^,^47^ For the sake of discussion, we begin by reporting on spectra collected in dilute HNO_3_ (0.05 M), then move to concentrated HNO_3_ (16 M), and conclude at the intermediate HNO_3_ concentration (4 M).

**Table 2:** The energy shift (Δ*E*_0_), bond distance (R), coordination number (CN), and Debye–Waller factor (*σ*^2^) fitting parameters used to model the room-temperature An^III^ L_3_-edge solution-phase EXAFS spectra from An^III^ cations (An^III^ = Cm^III^, Am^III^, Ac^III^) dissolved in HNO_3_ (0.05, 4, and 16 M). Data were additionally compared with the previously measured An^III^ aquo complexes.^12^,^21^,^46^,^47^ In each of our models, the structural amplitude reduction factors (*S*_0_^2^) were set to 0.9^*a*^

	Δ*E*_0_ (eV)	*R*_M–O_ (Å)	CN_bound oxygen_	*σ*_bound oxygen_^2^	*R*_M···N_ (Å)	CN_nitrogen_	*R*_M···O terminal oxygen_	*σ*_terminal oxygen_^2^
Cm aquo (0.25 M HCl),^46^^,^*	–13.0	2.450 ± 0.002	10.2 ± 0.3	0.009 (fixed)	—	—	—	—
Cm aquo (1 M HClO_4_),^47^^,^* fit 1	–2.0 ± 0.9	2.469 ± 0.007	7.0 ± 0.4	0.0071(8)	—	—	—	—
Cm aquo (1 M HClO_4_),^47^^,^* fit 2	–0.7 ± 0.7	2.470 ± 0.006	6 (fixed)	0.0053(2)	—	—	—	—
		2.63 ± 0.02	3 (fixed)	0.009 (2)				
Cm (HNO_3_, 0.05 M)	–5.5 ± 0.8	2.47 ± 0.01	9.6 ± 0.7	0.009(1)	—	—	—	—
Cm (HNO_3_, 4 M)	1.5 ± 1.3	2.45 ± 0.01	7 (fixed)	0.007(3)	2.93 ± 0.07	1 fixed	4.25 ± 0.03	0.006 (4)
		2.54 ± 0.05	2 (fixed)					
		2.63 ± 0.04	2 (fixed)					
Cm (HNO_3_, 16 M)	3.4 ± 1.1	2.49 ± 0.02	8.9 ± 2.2	0.009(2)	2.95 ± 0.02	4.1 ± 0.7	4.25 ± 0.02	0.010(3)
		2.64 ± 0.03	5.7 ± 1.3					
Am (0.25 M HCl),^46^^,^*	–8.7	2.480 ± 0.002	10.3 ± 0.3	0.009 (fixed)	—	—	—	—
Am (0.11 M HO_3_SCF_3_),^21^	–4.7 ± 0.9	2.48 ± 0.01	9.5 ± 0.9	0.0088(9)	—	—	—	—
Am (HNO_3_, 0.05 M)	–5.0 ± 1.0	2.47 ± 0.01	8.9 ± 0.8	0.008(1)	—	—	—	—
Am (HNO_3_, 4 M)	2.1 ± 1.3	2.46 ± 0.01	6 (fixed)	0.003(1)	2.98 ± 0.04	1 (fixed)	4.27 ± 0.04	0.003 (2)
		2.57 ± 0.03	2 (fixed)					
		2.67 ± 0.02	2 (fixed)					
Am (HNO_3_, 16 M)	3.3 ± 0.8	2.50 ± 0.01	7.7 ± 0.8	0.005(1)	2.97 ± 0.01	3.4 ± 0.7	4.26 ± 0.01	0.006(2)
		2.67 ± 0.01	5.4 ± 0.5					
Ac (0.11 M HO_3_SCF_3_),^12^	–3.9 ± 1.0	2.63 ± 0.01	10.9 ± 0.5	0.009 (fixed)	—	—	—	—
Ac (HNO_3_, 0.05 M)	–2.9 ± 1.6	2.63 ± 0.02	10.0 ± 0.9	0.009 (fixed)	—	—	—	—
Ac (HNO_3_, 4 M)	3.9 ± 2.0	2.61 ± 0.02	6 (fixed)	0.006(4)	3.24 ± 0.11	1 (fixed)	4.42 ± 0.05	0.003 (5)
		2.75 ± 0.04	2 (fixed)					
Ac (HNO_3_, 16 M)	4.4 ± 2.1	2.70 ± 0.02	12.9 ± 4.0	0.012(4)	3.20 ± 0.12	2.3 ± 1.7	4.42 ± 0.03	0.003(6)

^*a*^Additionally, *σ*_N_^2^ was fixed to σ_O_^2^ and CN_terminal oxygen_ set to CN_nitrogen_. Data found in the literature and marked with an asterisk (*) had *S*_0_^2^ values set to 1.

**Fig. 3:**
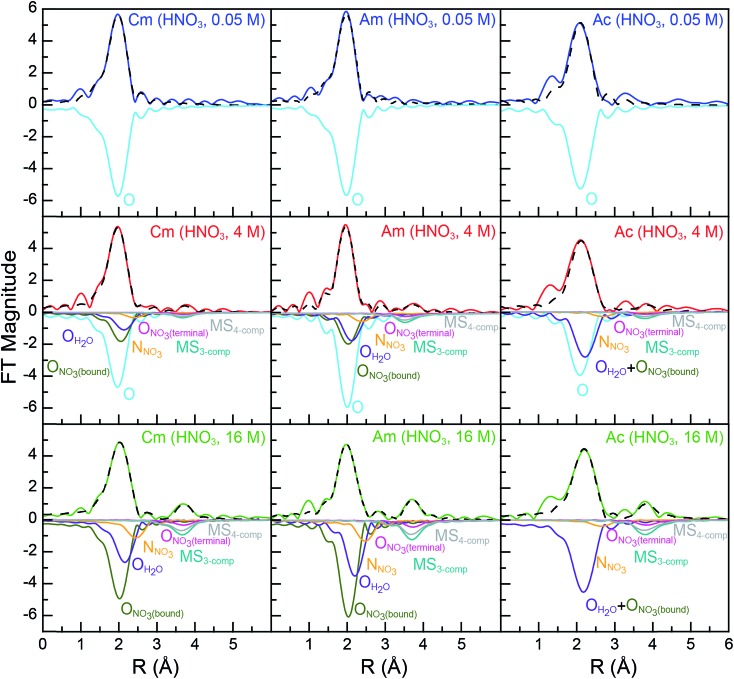
Fourier transform of room temperature solution-phase An^III^ L_3_-edge *k*^3^-EXAFS spectra of An^III^ (An = Cm^III^, left; Am^III^, middle; Ac^III^, right) cations dissolved in HNO_3_ (0.05 M, top, blue trace; 4 M, middle, red trace; 16 M, bottom, green trace). Fits to the data are shown as dashed black traces and scattering pathway contributions to the fit have been plotted inversely. The dilute HNO_3_ system (0.05 M) was modeled with a single H_2_O scattering pathway (cyan trace). The concentrated HNO_3_ system (16 M) was modeled with scattering pathways from O_H_2_O_ (purple trace), O_NO_3_(bound)_ (olive trace), N_NO_3__ (orange trace), and O_NO_3_(terminal)_ (pink trace). Additionally, two multiple scattering paths were included, a three competent pathway labeled MS_3-comp_ (An^III^ → O_NO_3_(terminal)_ → N_NO_3__ → An^III^; blue-green traces) and a four component pathway labeled MS_4-comp_ (An^III^ → N_NO_3__ → O_NO_3_(terminal)_ → N_NO_3__ → An^III^, grey trace). The intermediate HNO_3_ (4 M) data were modeled using a combination of fits for dilute HNO_3_ (0.05 M, cyan scattering pathway) and concentrated HNO_3_ (16 M; purple, olive, pink, orange, blue-green, and grey scattering pathways) spectra.

As shown in Fig. 2, all spectra collected from dilute HNO_3_ (0.05 M) solutions were best described by a single frequency whose amplitude in *k-*space (left, Fig. 2) dampened with increased energy. Best fits for the data (top, Fig. 3; Table 2) – those with the smallest residuals and lowest reduced chi-squared values – confirmed this superficial interpretation. The histogram of frequencies shown in the Fourier transform spectra (right, Fig. 2; top, Fig. 3) contained a single peak near *R* = 2 Å. As the frequency resolution ranged from 0.19 to 0.23 Å for Cm^III^, Am^III^, and Ac^III^, we refrained from attempting to resolve multiple M–O_H_2_O_ scattering pathways within this first water shell. Furthermore, the data quality was not sufficient for observing H_2_O molecules at longer distances, *i.e.* in the second and third hydration shells. Fitting the data with a single H_2_O shell revealed approximately nine water molecules for Cm^III^ (9.6 ± 0.7) and Am^III^ (8.9 ± 0.8) with equivalent M–O_H_2_O_ distances of 2.47(1) Å. These results agreed well with the literature values for Cm^III^ and Am^III^ aquo ions. The single crystal structure of the Cm^III^ aquo ion showed nine H_2_O ligands with an average Cm–O_H_2_O_ distance of 2.51(8) Å.^48^ Previous EXAFS measurements obtained from the Cm^III^ aquo ion in dilute HCl (0.25 M)^46^ and dilute HClO_4_ (1 M)^47^ showed 10.2 ± 0.3 oxygen atoms at 2.450(2) Å and 7.0 ± 0.4 oxygen atoms at 2.469(7) Å, respectively. Similarly, recent EXAFS studies characterized the Am^III^ aquo ion as having 9.5 ± 0.9 oxygen atoms at 2.48(1) Å (HO_3_SCF_3_; 0.11 M)^21^ and 10.3 ± 0.3 oxygen atoms at 2.480(2) Å (HCl; 0.25 M).^46^ A single crystal structure for the Am^III^ aquo ion has also been reported, showing nine H_2_O ligands with a 2.52(8) Å average Am–O_H_2_O_ distance.^48^

For the larger Ac^III^ cation, a longer Ac–O_H_2_O_ distance of 2.63(2) Å was observed. In comparison to the Cm^III^ and Am^III^ aquo ions described above, the larger Ac–O_H_2_O_ bond distance was statistically relevant. The Ac^III^ coordination number also seemed larger than those from Cm^III^ and Am^III^ with 10.0 ± 0.9 inner sphere H_2_O ligands. However, these values were equivalent when the measurement uncertainties were considered. The Ac–O_H_2_O_ bond distance and H_2_O coordination numbers were consistent with the only other data obtained on an Ac^III^ aquo ion,^12^ despite differences in the solution matrices; HNO_3_ (0.05 M) *vs.* HO_3_SCF_3_ (0.11 M). This previous analysis showed 10.9 ± 0.5 oxygen atoms at 2.63(1) Å. Additional confidence in these Ac–O_H_2_O_ distances was provided by comparison with previous Ac^III^ L_3_-edge EXAFS measurements made in HCl (11.7 M) solutions, which gave a 2.59(3) Å Ac–O_H_2_O_ distance.^21^ Overall, all of our An–O_H_2_O_ (An = Cm^III^, Am^III^, Ac^III^) distances were in agreement with the Shannon ionic radii.^42^ For example, subtracting the six coordinate ionic radii from the experimental M–O_H_2_O_ distances gave 1.50, 1.50, and 1.51 Å for Cm^III^, Am^III^, and Ac^III^, respectively. These values were bracketed by the calculated (1.67 Å) and crystallographically measured (1.38 Å) H_2_O ionic radii.^49^ In all of the An^III^ aquo spectra (HNO_3_, 0.05 M for Cm^III^, Am^III^, and Ac^III^; HO_3_SCF_3_, 0.11 M for Am^III^ and Ac^III^), there was no evidence of An^III^ aquo ion dimerization. No An^III^···An^III^ scattering pathways were detected nor was there evidence for short An^III^–OH interactions, which would result from hydrolysis. Hence, these data were consistent with previous EXAFS studies on An^III^ and Ln^III^ aquo ions,^12^,^50^–^54^ suggesting that Cm^III^, Am^III^, and Ac^III^ aquo ions existed primarily as discrete An^III^(H_2_O)_*x*_^3+^ species. However, EXAFS spectroscopy is relatively insensitive to dilute impurities, and dimeric species present at less than 10% of the total sample would be difficult to detect.^43^

Consistent with the Ac^III^ aquo L_3_-edge EXAFS spectra reported previously in dilute HO_3_SCF_3_, the data reported here contained a feature near 3.2 Å in the Fourier transform. To date, we have been unable to identify physically realistic models to explain these high-frequency oscillations. Given the instability of these features in various *k* ranges (7, 8, 9, 10 Å^–1^), at this time we believe their origin is not related to the Ac^III^ coordination chemistry and likely results from systematic artifacts related to the data quality. While not conclusive, this proposition was supported by the absence of this mysterious peak in the higher quality Cm^III^ and Am^III^ spectra, as long as one assumes analogous coordination chemistry exists for all three cations.

**Scheme 1:**
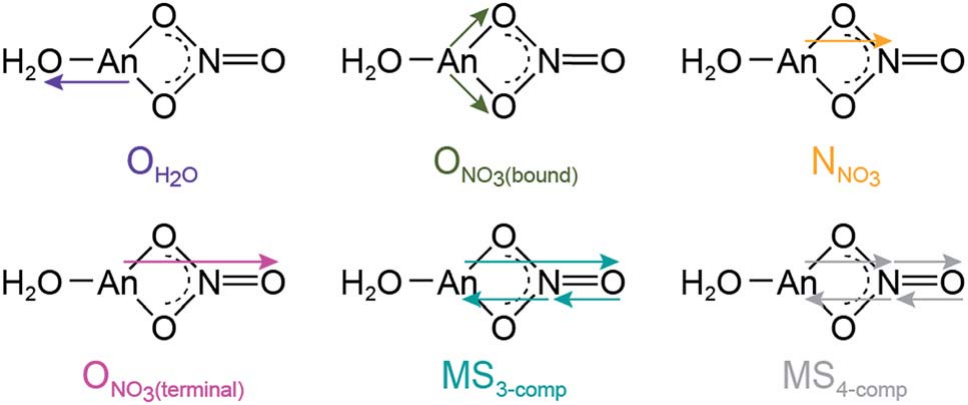
Scattering pathways deployed in fitting An L_3_-edge EXAFS data from An^III^ cations dissolved in 16 M HNO_3_.

Comparison between 0.05 and 16 M HNO_3_ offered the highest probability to identify differences in An^III^ speciation. Our approach to modeling these EXAFS data was consistent with previous models used to explain spectra from Ln^III^ and An^III^ cations dissolved in HNO_3_ (6.8 M,^55^ 13 M) (Scheme 1; bottom, Fig. 3). For Cm^III^ and Am^III^, there were two short oxygen scattering pathways. The shorter path was assigned to metal bound oxygen atoms from NO_3_^–^ ligands (O_NO_3_(bound)_, olive trace); meanwhile the other was attributed to a shell of oxygen atoms from the H_2_O ligands (O_H_2_O_, purple trace, Fig. 3; Scheme 1). However, because these designations resulted from calculations on static actinide nitrate molecules, we do not have high confidence in the rigidity of these assignments. For example, in solution, NO_3_^–^ and H_2_O ligand exchange could likely occur. For Ac^III^, the data were not sufficient to resolve the two O_H_2_O_ and O_NO_3_(bound)_ scattering pathways. Hence, in the Ac^III^ model, the O_H_2_O_ and O_NO_3_(bound)_ shells were combined (purple trace, bottom right, Fig. 3).

Consistent with An^III^–NO_3_^–^ binding was the presence of four higher frequency scattering pathways, characteristic of inner-sphere NO_3_^–^ ligands.^55^ There was a pathway at intermediate distances associated with the central nitrogen of the NO_3_^–^ anion, referred to as N_NO_3__ (orange trace, Fig. 3; *ca. R* = 2.5 Å). This shell was followed by the NO_3_^–^ terminal oxygen (O_NO_3_(terminal)_; pink trace, *ca. R* = 3 Å). Subsequently, between *ca.* 3 < *R* < 4.5 Å there were two linear multiple scattering pathways. There was the three component An^III^ → O_NO_3_(terminal)_ → N_NO_3__ → An^III^ (MS_3-comp_; blue-green trace) pathway and the four component An^III^ → N_NO_3__ → O_NO_3_(terminal)_ → N_NO_3__ → An^III^ (MS_4-comp_; grey trace) pathway (Scheme 1). Our attempts to model the data with bent multiple scattering pathways (*i.e.* An^III^ → O_NO_3_(bound)_ → N_NO_3__ → An^III^) or as dimers and oligomers (with An···An scattering paths) were unsuccessful. Best fits for the data contained η^2^-NO_3_^–^ (bidentate) ligands and were modeled using the following constraints. The O_NO_3_(terminal)_ amplitude (coordination number) was fixed to N_NO_3__, which in turn was allowed to converge. In addition, the N_NO_3__ and O_NO_3_(bound)_ Debye–Waller factors (*σ*^2^) were fixed to that associated with O_H_2_O_, as all three scattering pathways had similar frequencies and because these three scattering pathways combined to form a single peak in the Fourier transform. This constraint additionally minimized the number of free fitting parameters.

For Cm^III^, refinement of the model to experimental data showed 8.9 ± 2.2 O_NO_3_(bound)_ atoms at 2.49(2) Å. There were also 5.7 ± 1.3 O_H_2_O_ at 2.64(3) Å and 4.1 ± 0.7 N_NO_3__ atoms at 2.95(2) Å. The Cm^III^–O_NO_3_(terminal)_ distance was 4.25(2) Å (Table 2, Fig. 3). To determine the number of NO_3_^–^ ligands, two options existed involving either the N_NO_3__ coordination number or the O_NO_3_(bound)_ coordination number. Although, similar stoichiometries were obtained for both scenarios, reported here is a chemical formula based on N_NO_3_(bound)_ to facilitate comparison with the Ac^III^ data below. Overall, these data indicated that the average Cm^III^ species present in concentrated HNO_3_ (16 M) had a stoichiometry of Cm(NO_3_)_4.1±0.7_(H_2_O)_5.7±1.3_^(1.1±0.2)–^ with an overall coordination number of 13.9 ± 1.9. Stoichiometric self-consistency associated with the coordination number ratio for N_NO_3__, O_NO_3_(bound)_, O_NO_3_(terminal)_ (2 : 1 : 1) – as well as the magnitude of the multiple scattering pathways – provided additional confidence in our model.

The Am^III^ data in 16 M HNO_3_ resembled that from Cm^III^ (Table 2), albeit with slightly smaller uncertainties. For instance, this analysis showed that the average coordination numbers for all of the Am^III^ species present in concentrated HNO_3_ had 7.7 ± 0.8 O_NO_3_(bound)_ atoms at 2.50(1) Å, 5.4 ± 0.5 O_H_2_O_ atoms at 2.67(1) Å, 3.4 ± 0.7 N_NO_3__ atoms at 2.97(1) Å, and an Am–O_NO_3_(terminal)_ distance of 4.26(1) Å (Table 2, Fig. 3). Based on the N_NO_3__ and O_H_2_O_ values, the analysis suggested an average stoichiometry of Am(NO_3_)_3.4±0.7_(H_2_O)_5.4±0.5_^(0.4±0.1)–^ (mean coordination number of 12.2 ± 1.5). Again, the O_NO_3_(bound)_, N_NO_3__, and O_NO_3_(terminal)_ coordination numbers and magnitudes from the multiple scattering pathways were all self-consistent with this average stoichiometry.

Moving to the larger Ac^III^ ion had little effect on the overall coordination number, showing 12.9 ± 4 inner-sphere oxygen atoms. The average Ac(NO_3_)_2.3±1.7_(H_2_O)_8.3±5.2_^(0.7±0.5)+^ solution phase stoichiometry was (essentially) equivalent to that from Cm^III^ and Am^III^; however, the uncertainties associated with the Ac^III^ L_3_-edge measurements were larger. The presence of 2.3 ± 1.7 N_NO_3__ atoms at 3.20(12) Å and Ac^III^–O_NO_3_(terminal)_ atoms at 4.42(3) Å confirmed the presence of inner-sphere NO_3_^–^ ligands in HNO_3_ (16 M; Table 2, Fig. 3). The largest differences between the Cm^III^, Am^III^, and Ac^III^ L_3_-edge EXAFS data were associated with the Ac^III^ interatomic distances. As expected based on the ∼0.15 Å increase in Ac^III^ six coordinate ionic radii, the An^III^–O_H_2_O_ and An^III^–O_NO_3_(bound)_ distances increased by approximately 0.2 Å from Cm^III^ and Am^III^ to Ac^III^.

**Scheme 2:**
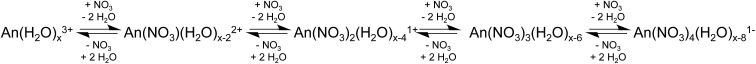
Various actinide nitrate speciation possibilities.

Because EXAFS spectroscopy probes all species in solution, it does not exclude An^III^ access to other stoichiometric ratios of NO_3_^–^ and H_2_O, Scheme 2. Instead, it provides an average signal from all of the molecules in the sample. In this context, good models of the data were only obtained with η^2^-NO_3_^–^ ligands (bidentate), which were consistent with many models previously reported for lanthanide and actinide EXAFS data.^50^,^56^–^58^ One notable exception was identified by Antonio and coworkers. These authors successfully identified monodentate η^1^-NO_3_^–^ binding for Ce^III^ in 3 M HNO_3_, a notably lower concentration than the 16 M HNO_3_ discussed here.^59^ Our attempts to introduce η^1^-NO_3_^–^ (monodentate) binding increased the An^III^ → N_NO_3__ and An^III^ → O_NO_3_(terminal)_ distances into unrealistic regions of the spectra where no intensity was present. Additionally, η^1^-NO_3_^–^ diminished the amplitude for linear multiple scattering pathways, giving an appreciable misfit between 3 < *R* < 4.5 Å in the Fourier transform. We interpret these results as suggesting that in 16 M HNO_3_ An^III^–η^2^-NO_3_ binding was preferred for Cm^III^, Am^III^, and Ac^III^ over monodentate modes, likely due to the chelation effect.^60^ Consistent with this observation were quantum calculations on M(NO_3_)_*x*_(H_2_O)_*y*_^3–*x*^ (M = Am^III^, Eu^III^) reported by Xi and coworkers.^61^ Their calculations predicted that η^2^-NO_3_ binding was preferred energetically in aqueous solutions, especially when the first coordination shell was sterically saturated. Xi's calculated 2.45 Å Am–[η^2^-O_NO_3_(bound)_] bond distance is in excellent agreement with our EXAFS results, lending confidence to our η^2^-NO_3_ binding model. As pointed out to us privately by Antonio, the larger An–NO_3_^–^ stability constants^62^ may be responsible for Cm^III^, Am^III^, and Ac^III^ preference for η^2^-NO_3_^–^ binding.^59^,^63^

**Fig. 4:**
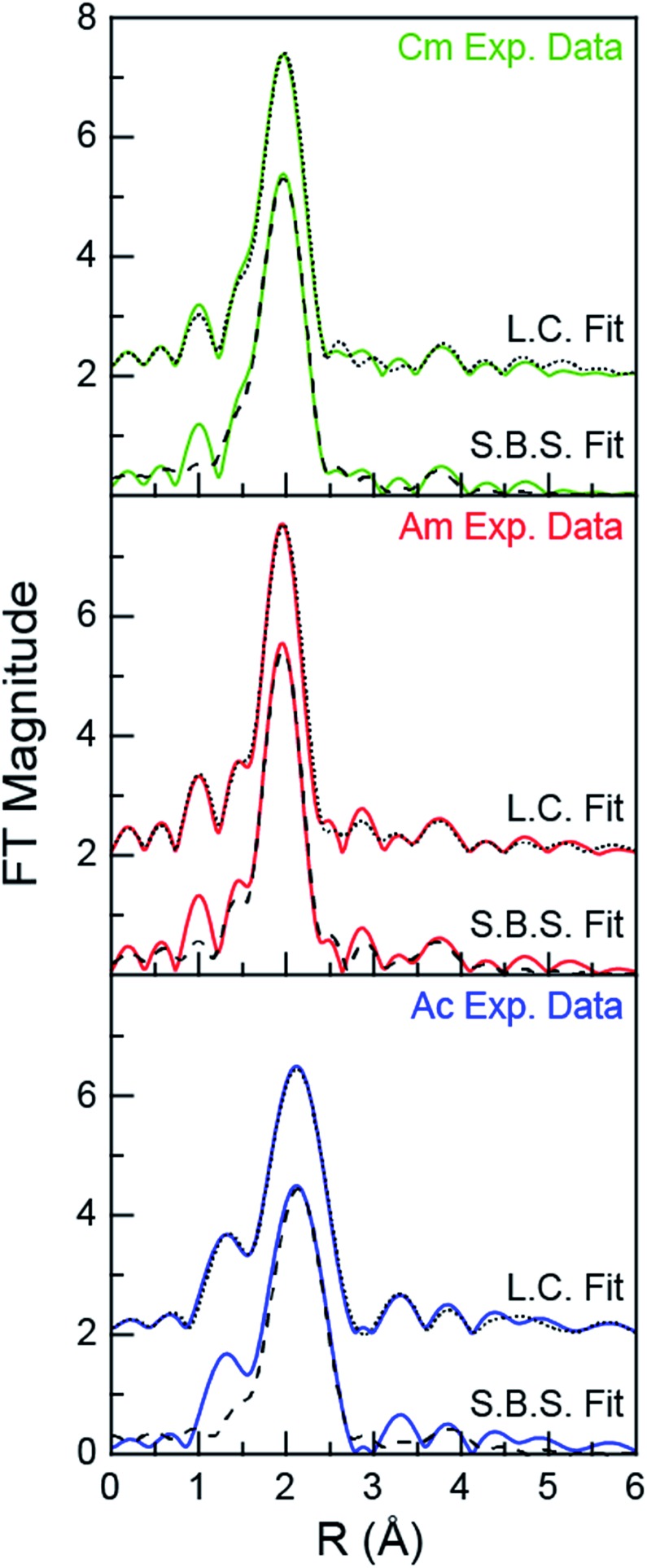
Fourier transform of the An^III^ L_3_-edge *k*^3^-EXAFS spectra of An^III^ dissolved in 4 M HNO_3_ (An = Cm^III^, top; Am^III^, middle; Ac^III^, bottom). Experimental data are shown as green, red and blue traces (for Cm^III^, Am^III^, and Ac^III^, respectively), linear combination fitting (L.C. Fit) results are shown as a dotted black trace, and shell-by-shell fitting (S.B.S. Fit) results are shown as a dashed black trace.

**Fig. 5:**
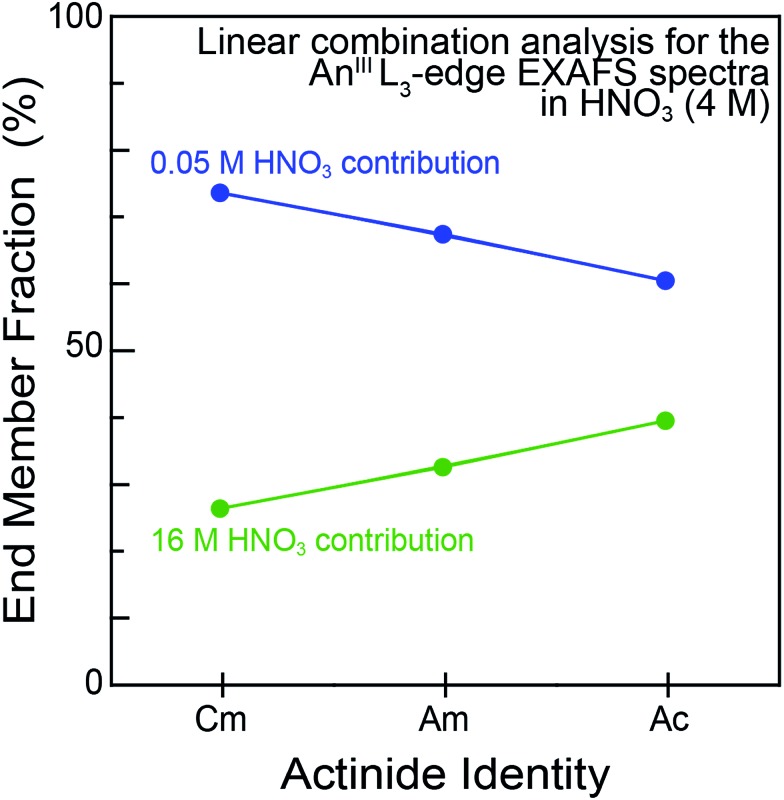
Linear combination analyses of the An^III^ L-edge EXAFS spectra of An^III^ cations dissolved in HNO_3_ (4 M; An^III^ = Cm^III^, Am^III^ and Ac^III^). The end member identities were obtained from fits to the Ac^III^ L_3_-edge EXAFS spectra from dilute (0.05 M; blue trace) *vs.* concentrated (16 M; green trace) HNO_3_ solutions.

For experiments conducted at the intermediate HNO_3_ concentration (4 M), an alternative fitting method was pursued. The initial model was generated from a linear combination of the two end members, namely An^III^ dissolved in 0.05 and 16 M HNO_3_. This fit (Fig. 4) suggested that the 4 M HNO_3_ Cm^III^ speciation could be described as containing 73.6(1.8)% of the Cm^III^ aquo ion and 26.4(1.8)% of the Cm(NO_3_)_4.1±0.7_(H_2_O)_5.7±1.3_^(1.1±0.2)–^ (Fig. 5). The slightly larger Am^III^ cation gave a similar ratio; 67.4(1.4)% of the Am^III^ aquo and 32.6(1.4)% Am(NO_3_)_3.4±0.7_(H_2_O)_5.4±0.5_^(0.4±0.1)–^. More substantial differences were observed when moving to the much bigger Ac^III^ ion. The analysis showed 60.5(1.4)% of the Ac^III^ aquo and 39.5(1.4)% of the Ac(NO_3_)_2.3±1.7_(H_2_O)_8.3±5.2_^(0.7±0.5)+^. These analyses assisted subsequent modeling efforts that used shell-by-shell methods, similar to those described above to fit the 0.05 and 16 M HNO_3_ spectra. The fitting routine for the HNO_3_ (4 M) data differed in that it included all of the scattering pathways used in the 0.05 and 16 M models. To keep the number of fitted parameters less than half of the total number of independent variables,^64^ the coordination numbers were fixed in accordance with the percentages determined from the linear combination analyses (Fig. 4 and 5). Under these conditions, variables associated with the interatomic distance (*R*) and Debye–Waller factors (*σ*^2^) were allowed to converge to reasonable values, as shown in Table 2. The good agreement of these shell-by-shell fits with the experimental data validated conclusions from the linear combination analyses, suggesting that the H_2_O and NO_3_^–^ coordination numbers were between those of the 0.05 and 16 M end-members.

## Outlook

The An^III^ L_3_-edge XAS results described herein represent a humble contribution to the growing body of knowledge associated with Cm^III^, Am^III^, and Ac^III^ coordination chemistry, aqueous speciation, and chemical reactivity.^10^–^21^,^65^–^69^ To most effectively communicate the significance of these results, we find it instructive to present the data within the context of some relevant studies reported for NO_3_^–^ binding of Cm^III^, Am^III^, and Ac^III^. For example, in the solid state, there are only two single crystal X-ray structures reported that contain Am–NO_3_ bonds. In these complexes the Am–O_NO_3__ distances ranged from 2.514 to 2.635(12) Å,^70^,^71^ which was in good agreement with our Am–O_NO_3__ results, 2.50(1) and 2.57(3). To date, we have been unaware of any Cm single crystal structures that contain Cm–NO_3_ bonds and no single crystal data of any kind have been reported for Ac. Solution-phase characterization using EXAFS spectroscopy is equally sparse. There is a report from Den Auwer on the Am(NO_3_)_3_(TEMA)_2_ complex,^72^ another by Girnt on Am(NO_3_)_*x*_(dmpbipy)_*x*_,^73^ one by Bremer involving Am(NO_3_)_*x*_(C5-BPP)_*x*_,^74^ and finally Ekberg investigated [Am(NO_3_)(CyMe_4_-BTBP)_2_]^2+^.^75^ These studies showed an average Am–O_NO_3__ distance of 2.49 ± 0.01 Å. For Cm, numerous measurements have been made on NO_3_^–^ species. These include (but are not limited to) time resolved laser fluorescence and luminescence measurements made on Cm^III^ in nitrate containing solutions,^11^,^76^,^77^ numerous studies documenting the extraction of Cm^III^ from HNO_3_ solutions,^78^–^81^ as well as thermal decomposition of Cm^III^(NO_3_)_3_.^82^,^83^ For Cm we are unaware of EXAFS measurements made in HNO_3_ solutions and for Ac the Ac–NO_3_^–^ interaction has not been characterized previously.

In the context of what is understood regarding complexation of Cm^III^, Am^III^, and Ac^III^ by NO_3_^–^ in aqueous media, the EXAFS results reported herein provide hard data that can be used broadly to assist applied and fundamental efforts that require An^III^ cations to be dissolved in HNO_3(aq)_. Our data suggested that Cm^III^, Am^III^, and Ac^III^ existed as aquo ions in dilute HNO_3_ matrices (0.05 M). These results agreed with the small An^III^–NO_3_ stability constants: ([An^III^–NO_3_]/[An^III^][NO_3_^–^]; log *K*, ionic strength = 1 M, 25 °C) 0.34 (Cm^III^),^84^ 0.25 ± 0.02 (Am^III^),^85^ and 0.1 (Ac^III^).^85^ Along these lines, Choppin and coworkers used Cm^III^ fluorescence to evaluate NO_3_^–^ complexation in aqueous solutions with varied HNO_3_ concentrations. In this study, moving from 0.1 to 13 M HNO_3_ decreased the number of bound H_2_O molecules, presumably accompanied by NO_3_^–^ complexation. A total of four H_2_O molecules were reportedly removed in 13 M HNO_3_, suggesting that a bis-nitrato [M(NO_3_)_2_(H_2_O)_5_]^1+^ complex had formed.^86^ Consistent with Choppin and coworkers' results,^86^ our EXAFS data showed that nitrate complexation for Cm^III^, Am^III^, and Ac^III^ increased with increasing HNO_3_ concentration. In 4 M HNO_3_, we observed approximately one inner sphere NO_3_^–^. Moving past Choppin and Coworkers' 13 M HNO_3_ to concentrated HNO_3_ (16 M), increased the number of coordinated NO_3_^–^ ligands, ranging from 4.1 ± 0.7 for Cm^III^, to 3.4 ± 0.7 for Am^III^, and 2.3 ± 1.7 for Ac^III^. It is interesting that the NO_3_^–^ coordination numbers seemed to decrease with increasing metal ionic radius. While tempting to correlate these results with the stability constants referenced above and with the Lewis acidity for the An^III^ cations, we refrain since the NO_3_^–^ coordination numbers were equivalent when the uncertainties for the measurements were considered.

In terms of structural characterization, the Ac^III^–O_H_2_O_ and Ac^III^–O_NO_3_(bound)_ bond distance measurements represent another impactful component of this manuscript. Prior to these experiments, there were two reported Ac–O_H_2_O_ bond distances, both measured by solution-phase Ac^III^ L_3_-edge EXAFS spectroscopy. One was in concentrated HCl (11 M) solutions (2.59 ± 0.03 Å)^21^ and the other in dilute HO_3_SCF_3_ (0.11 M; 2.63 ± 0.01 Å).^12^ Contributed here are three additional Ac–O_H_2_O_ measurements; 2.63 ± 0.02 (0.05 M HNO_3_), 2.61 ± 0.02 (4 M HNO_3_), and 2.70 ± 0.02 (16 M HNO_3_). This brings the total number of reported Ac–O_H_2_O_ bond distances to five, averaging 2.63 ± 0.04 Å (error reported as the standard deviation of the mean, 1 *σ*). Their consistency provides confidence in the accuracy of these Ac^III^ L_3_-edge EXAFS measurements. In terms of NO_3_^–^ complexation, these results are also exciting as they represent the first Ac^III^–NO_3_^–^ interaction observed spectroscopically. Although the Ac^III^–O_NO_3__ distance was not resolved from the inner-sphere Ac^III^–O_H_2_O_ interaction, the Ac^III^–O_NO_3__ bond length can be indirectly inferred based on the measured Ac^III^–N_NO_3__ distance. For example, the Ac–O_NO_3_(bound)_ distance can be calculated using the cosine rule; assuming an average N–O distance of 1.31 Å and an average Ac–N–O angle of 113°.^57^ This analysis gives an Ac^III^–O_NO_3__ distance of 2.70 ± 0.10 Å.

In terms of fundamental exploratory science, the chemistry of Cm^III^, Am^III^, and Ac^III^ presents uncharted landscapes in comparison to many other elements in the periodic table. Unique safety hazards and limited access to sizable quantities of material represent significant technical challenges faced during experimental studies of these elements. Even interactions with common ligands – such as the An–H_2_O and An–NO_3_ bonds – are poorly defined. On top of scientific curiosity is the need to support innovation for An^III^ processing. This need includes developing advanced nuclear fuel cycles, medical isotope production, and targeted alpha therapy. It seems likely that our approach for characterizing An–NO_3_ and An–H_2_O (An = Cm^III^, Am^III^, Ac^III^) interactions using An^III^ L_3_-edge EXAFS can be broadly applied to other An^III^–ligands interactions, which are equally relevant for nuclear processing and medical applications. We hope that the results presented herein will provide insight aiding our current efforts – as well as those associated with other researchers embarking on their own fundamental and applied scientific campaigns – to solve complicated technical problems associated with Cm^III^, Am^III^, and Ac^III^.

## Experimental section

### General consideration

**Caution!** The ^246/248^Cm [*t*_1/2_ = 4706(40) years/3.48(6) × 10^5^ years],^87^^243^Am [*t*_1/2_ = 7364(22) years],^87^ and ^227^Ac [*t*_1/2_ = 21.772(3) years]^87^ isotopes present serious health threats due to their (as well as their daughters) direct neutron-, α-, β-, and γ- emissions of their radioactive daughters. Hence, all studies that involved uncontained manipulations were conducted in a radiation laboratory equipped with HEPA filtered hoods, continuous air monitors, negative pressure gloveboxes, and monitored equipment appropriate neutron-, α-, β-, and γ-particle detection. All free-flowing solids were handled within negative pressure gloveboxes equipped with HEPA filters. The ^246/248^Cm, ^243^Am, and ^227^Ac isotopes were supplied by the United States Department of Energy Office of Science Isotope Program in the Office of Nuclear Physics. Chemically pure Cm^III^, Am^III^, and Ac^III^ stock solutions were prepared as previously described.^12^,^20^,^31^ Optima grade nitric acid was obtained commercially (Fisher Scientific). Water was purified to 18.2 MΩ cm^–1^ resistivity using Thermo-Scientific Barnstead Nanopure or Millipore Nanopure water purification systems. For Ac^III^, the water was further purified by using a Teflon distilling apparatus.

### Sample preparation

Three solution-phase XAS samples were prepared for each element. The first was prepared in 0.05 M HNO_3_, the second in 4 M HNO_3_ and the third in 16 M (concentrated) HNO_3_. To prepare the samples, aliquots from purified Cm^III^ (0.5 mg; 2.02 μmol), Am^III^ (0.5 mg; 2.06 μmol), and Ac^III^ (28 μg; 0.123 μmol) stock solutions were transferred to conical glass vials. The aqueous solution was removed by heating the samples on a hot plate at around 110 °C under a flow of argon gas until soft dryness was achieved. The residue was dissolved in aqueous nitric acid of desired molarity. Each sample was boiled and dissolved three times to ensure that the HNO_3_ concentration was actually 0.05, 4, or 16 M. The solution volumes for each sample were 0.5 mL (Am), 0.5 mL (Cm), and 0.3 (Ac). The resulting solutions were transferred to an XAS holder.

### Radiological containment for XAS samples

The XAS holders and handling procedures provided adequate containment (three layers) and administrative/engineering controls that guarded against release of radiological material during shipment and during data acquisition. The holder consisted of a plastic body with a 5 mm well for Cm^III^ and Am^III^ and a 2 mm well for Ac^III^ equipped with a set of Teflon windows (1 mil) and a Kapton window (1 mil). Solutions were introduced into the holder through an injection hole sealed with a Teflon gasket that was held in place by an aluminum plate. This primary holder was then held within a secondary container, which in turn was held within the tertiary container. The secondary and tertiary containers are best described as a set of nested aluminum holders equipped with Kapton windows (2 mil) and rubber gaskets.

### XAS data acquisition

The actinide L_3_-edge XANES and EXAFS measurements were made at the Stanford Synchrotron Radiation Lightsource (SSRL) under dedicated operating conditions (3.0 GeV, 5%, 500 mA) on end station 11-2. This beamline was equipped with a 26-pole and a 2.0 tesla wiggler. Using a liquid nitrogen-cooled double-crystal Si(220) (*Φ* = 0° for Cm^III^; *Φ* = 90° for Am^III^ and Ac^III^) monochromator and employing collimating and focusing mirrors, a single energy was selected from the incident white beam. Vertical acceptance was controlled by slits positioned before the monochromator. For Cm^III^, the monochromator crystals were 35% detuned. Meanwhile, Am^III^ and Ac^III^ L_3_-edge measurements were conducted with the monochromator crystals fully-tuned. For these experiments, higher harmonics from the monochromatic light were removed using a 370 mm Rh coated harmonic rejection mirror. The Rh coating was 50 nm with a 20 nm seed coating and the substrate was Zerodur. The harmonic rejection cut-off was set by the mirror angle, controlling which photons experience total external reflection. The samples were attached to the beamline 11-2 XAS rail. The rail was equipped with three ionization chambers through which nitrogen gas was continually flowed. One chamber was positioned before the sample holder to monitor the incident radiation (*I*_0_, 10 cm). The second chamber was positioned after the sample holder, such that sample transmission (*I*_1_, 30 cm) could be evaluated against *I*_0_, while a third chamber (*I*_2_, 30 cm) was positioned downstream from *I*_1_ so that the XANES of a calibration foil could be measured *in situ* during the XAS experiments against *I*_1_. All actinide L_3_-edge XAS spectra were measured by monitoring sample fluorescence against the incident radiation (*I*_0_). The detector was positioned 90° to the incident radiation (I_0_). For Cm^III^ and Am^III^ a Lytle detector, equipped with Soller slits and Sr (3 absorption lengths) filters were used. For Ac^III^ measurements, a solid-state 100-element Ge detector was used. This detector was windowed on the Ac^III^ Lα_1_-emission line (12.652 keV). High-energy contributions to the fluorescence signal were removed using a bromine filter (6 absorption lengths). Using a Se filter, detector dead time was characterized approximately 400 eV above the Se K-edge by defining the detector response from 0 to ∼70% dead (windowed counts of the emission line *versus* the total of incoming counts in the solid-state detector).

### XAS data analysis

Data manipulation and analysis was conducted as previously described.^21^,^43^ All calibration spectra were measured *in situ*. The Cm^III^ and Am^III^ spectra were calibrated to the energy of the absorption peak maximum of a Zr foil (18 013.3 eV (ref. 88)). The actinium sample data were dead time corrected and calibrated to the energy of the first inflection point of a rubidium(ii) chloride, RbCl, pellet diluted with boron nitride (BN) to a 1 absorption length thickness. The energy for the first inflection point for RbCl was determined in comparison to the Bi L_2_-edge of a bismuth foil (15 711 eV) to be 15 203.8 eV.

The XAS data were analyzed by fitting a line to the pre-edge region, which removed the background from experimental data in the spectra. Then a third order polynomial fit was chosen for the post-edge region. The difference between pre and post edge lines was set to unity at the first inflection point, normalizing the absorption jump to 1.0. Samples were measured for several hours resulting in the collection of multiple scans. The EXAFS data were analyzed by either shell-by-shell fitting methods using IFEFFIT software^88^ and FEFF8 calculations^44^,^45^ or linear combination analyses (IFEFFIT).^88^ Atomic coordinates for the FEFF8 calculations were obtained by geometry optimizations generated from DFT calculations (see below). Data were fit over the following ranges; for curium and americium 2.7 < *k* < 11 Å^–1^ and 1.1 < *R* < 4.5 Å (to 3 Å for 0.05 M) and for actinium 2.7 < *k* < 9.5 Å^–1^ and 1.25 < *R* < 4.5 Å.

### DFT calculations

All DFT calculations were performed with ORCA version 4.0.1.^89^ Calculations utilized the PBE functional,^90^ the SARC-ZORA-TZVP^91^,^92^ and def2 (ref. 93) basis sets, and the D3 dispersion corrections.^94^,^95^ Coordinates of the DFT optimized structures are given in the ESI.†

## Conflicts of interest

There are no conflicts to declare.

## Supplementary Material

Supplementary information
