# Supplementary material for: The coordination chemistry of CmIII, AmIII, and AcIII in nitrate solutions: an actinide L3-edge EXAFS study
Source: Chem Sci. 2018 Aug 1;9(35):7078–90. doi: 10.1039/c8sc02270d (PMC6137438; doi:10.1039/c8sc02270d)
Supplement: Supplementary file 1 [file SC-009-C8SC02270D-s001.pdf]

Electronic Supplementary Material (ESI) for Chemical Science.
This journal is © The Royal Society of Chemistry 2018

A Series of Dithiocarbamates for Americium, Curium, and Californium.

Samantha K. Cary,[a‡] Jing Su,[a‡] Shane S. Galley,[b‡] Thomas E. Albrecht-Schmitt,[b*] Enrique R. Batista,[a*] Maryline G. Ferrier,[a] Stosh A. Kozimor,[a*] Veronika Mocko,[a] Brian L. Scott,[a] Cayla E. Van Alstine,[b] Frankie D. White,[b] Ping Yang[a*]

[‡] Cary, Su, and Galley contributed equally to this work.

[a] Los Alamos National Laboratory, Los Alamos, New Mexico 87545

[b] Florida State University, Department of Chemistry and Biochemistry, Tallahassee, Florida 32306

LA-UR-18-22699

Dedicated to Professor Richard A. Andersen for his creative and inspirational contributions to f-element chemistry.

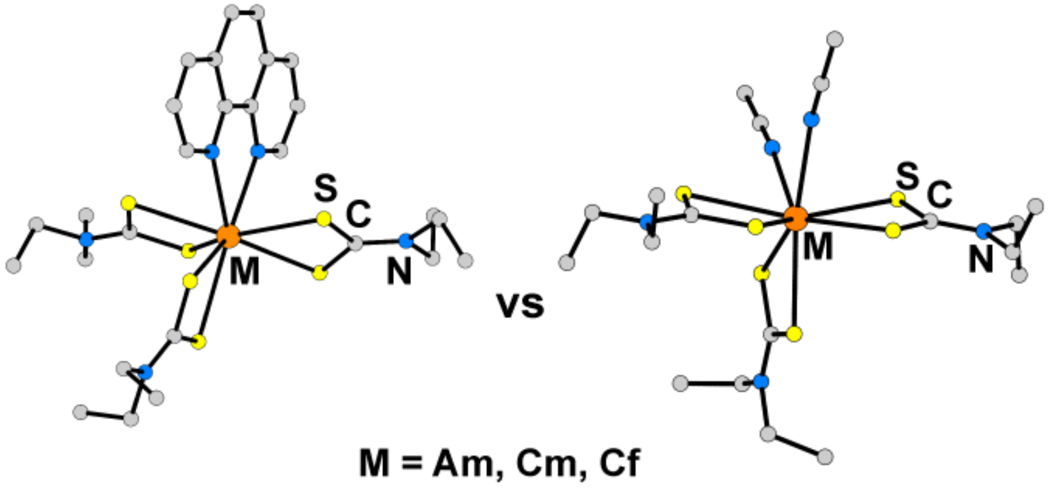

ABSTRACT: Characterizing how actinide properties change across the *f*-element series is critical for improving predictive capabilities and solving many nuclear problems facing our society. Unfortunately, it is difficult to make direct comparisons across the 5*f*-element series because so little is known about trans-plutonium elements. Results described herein help to address this issue through isolation of $An(S_2CNEt_2)_3(N_2C_{12}H_8)$ (Am, Cm, and Cf). These findings included the first single crystal X-ray diffraction measurements of Cm–S (mean of 2.86 ± 0.04 Å) and Cf–S (mean of 2.84 ± 0.04 Å) bond

distances. Furthermore, they highlight the potential of $An(S_2CNEt_2)_3(N_2C_{12}H_8)$ for providing a test bed for comparative analyses of actinide versus lanthanide bonding interactions.

Introduction

Identifying how elemental properties change across the periodic table provides basic insight for rationalizing complicated phenomena and advancing technical areas throughout the physical and biological sciences. Understanding periodic trends such as reactivity, conductivity, magnetism, electronegativity, solid-state structures, electronic interactions, atomic radii, ionization energies, etc. form the basis of modern day chemistry and physics. In fact, understanding periodicity is often taken for granted as a predictive tool when designing experiments to solve complicated problems. For atomic numbers greater than 92 (transuranic) the situation is different. For these elements, many periodic trends have yet to be established, owing to challenges associated with obtaining, safely handling, and studying these highly radioactive elements. As a result, transuranic behavior is often inferred from the large number of thorium and uranium studies or by extrapolating from their lanthanide congeners, whose chemistry is relatively well established by comparison. Unfortunately, the validity of these comparisons is precarious, as there is an absence of structurally characterized transuranic compounds that can be directly compared with uranium, thorium, or the lanthanide elements. Transforming the uncharted chemistry and physics for these elements into well-defined concepts that augment predictive capability represents one of the most daunting challenges in modern day exploratory science. Such advances are essential for solving applied and technical problems facing society today. For example, increasing fundamental understanding of transuranic chemistry is critical for managing fate and transport of radioactive elements in the environment, establishing safe methods to store and dispose of nuclear waste, solving partitioning and transmutation issues associated with spent nuclear fuel, and developing advanced nuclear fuel cycles.[1,2]

Inspired by recent advances at the extreme end of the *f*-element series,[3-6] we report herein the synthesis and characterization of a new family of trans-plutonium compounds, namely actinide(III) tris-diethyl(dithiocarbamate) 1,10-phenanthroline, $An(S_2CNEt_2)(N_2C_{12}H_8)$ (An = Am, Cm, and Cf). Additionally, we developed an efficient method to recycle small quantities (mg) of the rare and valuable ^{248}Cm isotope from experimental campaigns for future use. Accompanying these results were the first single crystal X-ray diffraction measurements of Cm–S and Cf–S bond distances. Isolation of these structurally similar compounds provided a well-defined and ubiquitous platform for validating computational results and theoretical concepts. Overall, the results generated insight into bonding trends across the actinide series and between lanthanide and actinide congeners, with particular emphasis on better characterizing the complicated $An–N_{N2C12H8}$ interaction.

Results and Discussion

Synthesis. Chemically pure ^{243}Am, ^{248}Cm, and ^{249}Cf stock solutions were prepared using well-established dissolution/separation procedures reported previously.[7-9] The Am^{III} stock solution was prepared by dissolving AmO_2 in nitric acid (HNO_3). For ^{248}Cm and ^{249}Cf experiments, the An^{III} cations were recovered from residues that had been used in previous experimental campaigns (Scheme 1). For instance, after dissolving the Cm^{III} sample in hydrochloric acid (HCl; 6 M), Cm^{III} was purified from inorganic and organic contaminants using a series of precipitations and ion exchange chromatography steps. Initial purification began by precipitating curium(III) fluoride with hydrofluoric acid (HF).[7,9] Next, highly insoluble curium(III) fluoride was dissolved in a combination of boric acid (H_3BO_3) and HCl. After an ammonium hydroxide (NH_4OH) precipitation, a more rigorous purification of the ^{248}Cm sample was achieved using ion exchange chromatography. This involved adsorbing Cm^{III} to a cationic exchange resin

(AG 50 x 8; 100-200 mesh, 2 mL of resin) in dilute HCl (0.1 M), washing with dilute HCl (0.1 M), and eluting Cm^{III} with concentrated HCl (12 M). Characterizing the eluent using optical spectroscopy (*e.g.* absorption and fluorescence) was not useful because of the low ^{248}Cm concentrations (10 mg total). Instead, the Cm^{III} elution profile was followed by stippling small volumes (one drop) of the eluted fraction onto Pyrex slides. After the samples dried under air, gross ^{248}Cm α-activity was quantified by analyzing each slide using a Ludlum 3939E α-, β-, γ-stationary survey instrument (for low activity samples) or a hand held portable α-survey meter (Ludlum 139) for high activity samples. Afterwards, stippled ^{248}Cm was recovered by soaking the slides in HCl (6 M) and set aside to be reprocessed at a later date.

The ^{249}Cf recovery procedure differed from that described for ^{248}Cm in two major ways. First, owing to constraints at the time of reprocessing, it was not possible to centrifuge the ^{249}Cf sample. As a result, the HF precipitation was not included. Second, because aspects of the ^{249}Cf sample history were well defined, we suspected appreciable amounts of iron contamination. Hence, peroxide (H_2O_2) and heat were used to convert Fe^{II} to Fe^{III}. Subsequently, the ferric contaminant was removed by passing the sample (in concentrated HCl) through an anion exchange resin (AG-MP1; 50 – 100 mesh, 2 mL). Under these conditions the Cf^{III} passed through the resin during the column load and in the first few column washes (6 M HCl). Next, a cation exchange resin (AG 50 x 8; 100-200 mesh, 5 mL) was used to generate a clean Cf^{III} stock solution in HCl (6 M). The chromatogram was characterized by monitoring the ^{249}Cf γ-ray emissions at 333.37(2) KeV [15.0(6)%] and 388.17(2) KeV [66.0(24)%].[10] Although the ^{248}Cm and ^{249}Cf purification procedures reported herein – as well as the ^{243}Am procedure reported previously[8] – provided adequate purity for preparing the $An(S_2CNEt_2)_3(N_2C_{12}H_8)$ complexes described below, these procedures cannot be blindly applied to every +3 actinide residue. Additional separation steps may be required to remove exotic contaminants.

Well-established synthetic procedures developed by *Stoll* and *Coworkers* for the lanthanide(III) tris-diethyl(dithiocarbamate) 1,10-phenanthroline complexes, $Ln(S_2CNEt_2)_3(N_2C_{12}H_8)$ (Ln = La, Pr, Sm, Eu, Gd, Tb, Dy),[11,12] were modified to prepare $An(S_2CNEt_2)_3(N_2C_{12}H_8)$ (An = Am, Cm, Cf^{III}). As observed for $Ln(S_2CNEt_2)_3(N_2C_{12}H_8)$, both Am^{III} and Cf^{III} nitrates, $An(NO_3)_3{\cdot}X(H_2O)$, reacted with three equivalents of diethyl-dithiocarbamate, $S_2CNEt_2{}^{1-}$, and one equivalent of 1,10-phenanthroline, $N_2C_{12}H_8$, in acetonitrile

$$\begin{array}{l} An(NO_3)_3 \cdot X(H_2O) \\ \quad + 3\,Z(S_2CNEt_2) \\ \qquad\qquad + N_2C_{12}H_8 \end{array} \longrightarrow \begin{array}{l} An(S_2CNEt_2)_3(N_2C_{12}H_8) \\ \qquad\qquad + 3\,X\,H_2O \end{array} \qquad \textbf{(1)}$$

$$\begin{array}{c} An = Am^{III}, Cm^{III}, Cf^{III} \\ Z = NH_2Et_2{}^{+}, Na^{+} \end{array}$$

(MeCN) at room temperature under air to form $An(S_2CNEt_2)_3(N_2C_{12}H_8)$, Eq 1. This reaction involved An^{III} coordination by neutral $N_2C_{12}H_8$, loss of outer-sphere H_2O, and substitution of $NO_3{}^{1-}$ for $S_2CNEt_2{}^{1-}$. Although the $Am(S_2CNEt_2)_3(N_2C_{12}H_8)$ and $Cf(S_2CNEt_2)_3(N_2C_{12}H_8)$ complexes were prepared using $(NH_2Et_2)(S_2CNEt_2)$, successful ionic salt metathesis of $NO_3{}^{1-}$ for $SCNEt_2{}^{1-}$ did not seem reliant on the cation's identity. For example, the Cm^{III} complex was generated using NaS_2CNEt_2.

Structure. Crystals suitable for single crystal X-ray diffraction of $An(S_2CNEt_2)_3(N_2C_{12}H_8)$ (An = Am, Cm, Cf) were grown over the course of an hour. All of the $An(S_2CNEt_2)_3(N_2C_{12}H_8)$ complexes crystalized in the monoclinic space group $P2_1/c$ (Figure 1). However, MeCN solvent molecules co-crystalized alongside $Cf(S_2CNEt_2)_2(N_2C_{12}H_8)$, causing distinct long-range crystalline order and imparting a slightly different unit cell. Despite these variations, the inner coordination spheres for all three trans-plutonium complexes were similar in that they consisted of 6 sulfur atoms and 2 nitrogen atoms. Analysis using the *SHAPE8* program suggested two geometries, with the coordination environment around Am^{III} and Cm^{III} being best described as square antiprisms and Cf^{III} as a dodecahedron.[13-15]

Crystallization of $An(S_2CNEt_2)_3(N_2C_{12}H_8)$ enabled the first single crystal X-ray diffraction measurements of An–S bond distances for Cm^{III} (mean Cm–S = 2.86 ± 0.04 Å) and Cf^{III} (mean Cf–S = 2.84 ± 0.04 Å), and only the second example of Am–S distances,[8] to be made. For convenience to the reader, the structural data has been summarized in Figure 2 and Table S1 and S2 by plotting the mean An–S and An–N distances versus metal ionic radii (uncertainty reported as standard deviation of the mean, 1σ). To place the data in a broader context, Ln–S and Ln–N bond distances from $Ln(S_2CNEt_2)_3(N_2C_{12}H_8)$ (Ln = Sm,[11] Eu[11], Gd,[5b] Dy[11]) were also included (Figure 2). In both cases and for all metals – aside from Cf – there was a near linear relationship between the measured bond distances and the metal ionic radii, with slopes of 0.76 ± 0.12 (M–S) and 1.11 ± 0.10 (M–N). Although the 1.53± 0.09 Å *y*-intercept for the M–N data was slightly higher than the 1.46 Å ionic radius of N^{3-}, these values overlapped when the measurement uncertainty was considered. Similarly, the 2.14 ± 0.11 Å *y*-intercept determined from the M–S data was larger than the expected for the S^{2-} ionic radius (1.84 Å).

Structural metrics from $Cf(S_2CNEt_2)_3(N_2C_{12}H_8)$ were excluded from the linear regression analyses discussed above because the average Cf–S and Cf–N distances were slightly shorter than expected. For example, Cf–ligand bond distances should be bracketed by those from Eu^{III} and Sm^{III}, based on the Cf^{III} ionic radii of 0.95 Å.[16] This was not the case. Even the longest Cf–S and Cf–N distances were shorter than the shortest Eu^{III} and Sm^{III} bond lengths. It was tempting to associate these minor variants as indicators of increased covalency in Cf–ligand bonding, relative to the other +3 metals. However, these structural deviations were slight and likely marginally relevant statistically. Rather than overstating the implications of these subtle bond distance deviations, we instead use these results as motivation for future efforts to uncover more substantial evidence of covalent Cf–S and Cf–N bonding using suitable spectroscopic and computational methods.

Optical Spectroscopy. Absorption spectra from single crystals of $An(S_2CNEt_2)_3(N_2C_{12}H_8)$ (An = Am, Cm, Cf) were collected using a Craic microspectrophotometer (Figure 3). All three spectra were dominated by broad high-energy (>20,000 cm^{-1}; <500 nm) absorptions. Given the commonality of these features in the ^{234}Am, ^{248}Cm, and ^{249}Cf spectra – as well as with the previously reported $Ln(S_2CNEt_2)_3(N_2C_{12}H_8)$ spectra – these absorption features were likely associated with $\pi \rightarrow \pi^*$ transition from the $N_2C_{12}H_8$ and S_2CNEt_2 ligands.[11] Superimposed on the tails of these intense transitions and at lower-energy (≤ 24,000 cm^{-1}) were Laporte forbidden $5f \rightarrow 5f$ transitions associated with the ^{243}Am, ^{248}Cm, and ^{249}Cf in the +3 oxidation state. Unfortunately, because of the nature of the solid-state absorption measurement, spectral intensities could not be compared. However, they did provide quantitative information regarding peak energy. For example, the main $Am(S_2CNEt_2)_3(N_2C_{12}H_8)$ absorption lines were measured at 19,207 cm^{-1} ($^7F_0 \rightarrow {}^5L_6$) and 12,260 cm^{-1} ($^7F_0 \rightarrow {}^7F_5$). Comparisons with the only other sulfur containing americium compound reported in the literature to date, $Am[S_2P(^tBu_2C_{12}H_6)]^{1-}$,[8] showed virtually no change for the $^7F_5$ transitions, only 36 cm^{1-} (2 nm) from 12,225 cm^{-1}. More pronounced (247 cm^{1-}; 7 nm) energy shifts were observed for the $^5L_6$ transitions, which was reported to be 19,455 cm^{-1}. This 247 cm^{-1} energy shift is larger then we expected. To calibrate the reader, consider that Am^{III} UV-vis measurements made on non-sulfur containing compounds show smaller energy variations, around or less than 100 cm^{-1}, *e.g.* $Am(C_5H_5)_3$, AmX_3 (X = Cl, Br, I), $Am_2(HPO_3)_3(H_2O)$, and $Am[B_9O_{13}(OH)_4]\cdot H_2O$ series.[10, 17, 18] At this stage, the origin of these energy shifts is difficult to interpret, as there are too few solid state UV-vis spectra available for comparative analysis. However, these types of absorption measurements are likely good indicators of electronic communication between the americium and the ligand valence orbitals, and potentially relevant for the other trans-plutonium elements.

While there are numerous solution phase Cm^{III} and Cf^{III} UV-vis spectra,[19-23] where speciation can be difficult to characterize, the number of solid state UV-vis data sets collected from well defined crystalline

solids are limited. Hence, it was challenging to identify spectra where peak energies could be directly compared with the $Cm(S_2CNEt_2)_3(N_2C_{12}H_8)$ and $Cf(S_2CNEt_2)_3(N_2C_{12}H_8)$ measurements. As such, the single crystal spectra from $Cm(S_2CNEt_2)_3(N_2C_{12}H_8)$ and $Cf(S_2CNEt_2)_3(N_2C_{12}H_8)$ represent humble contributions to the limited Cm^{III} and Cf^{III} single crystal UV-vis spectral archive; $M(HDPA)_3$ (M = Cm, Cf)[3], $Cm(H_2DPA)(HDPA)(H_2O)_2Cl$[5], $Cm_2[B_{14}O_{20}(OH)_7(H_2O)_2Cl]$[24], $M_4[B_{16}O_{26}(OH)_4(H_2O)_3Cl_4]$ (M = Cm, Cf)[25], $Cf[B_6O_8(OH)_5]$[4]. Based on previous spectral interpretations,[21-23, 26] and from the perspective of the free ion, we interpreted the absorption features from $An(S_2CNEt_2)_3(N_2C_{12}H_8)$ as resulting from exciting either the Cm^{III} $^8S_{7/2}$ or the Cf^{III} $^6H_{15/2}$ ground states to the excited states shown in Figure 3.[27,20, 28, 29[9b, 14]20, 28, 29[9b, 14][9b, 14][9b, 14]] Somewhat unexpectedly, excitation of $Cf(S_2CNEt_2)_3(N_2C_{12}H_8)$ and $Am(S_2CNEt_2)_3(N_2C_{12}H_8)$ at 23,810 and 27,397 cm^{-1} did not produce detectible Am^{III} or Cf^{III} fluorescence.[3, 4] In contrast, $Cm(S_2CNEt_2)_3(N_2C_{12}H_8)$ showed strong $5f \rightarrow 5f$ ($^6D_{7/2} \rightarrow {}^8S_{7/2}$) fluorescence at 16,367 cm^{-1} (611 nm) when excited at 23,810 and 27,397 cm^{-1}, characteristic of Cm^{III} (Figure S2).

Computational Studies. To provide insight into what factors contributed to stability of the An^{III}–$N_2C_{12}H_8$ interaction, thermodynamic and chemical bonding properties were evaluated using DFT calculations for $An(S_2CNEt_2)_2(N_2C_{12}H_8)$ versus a hypothetical series of complexes that contained more simple nitrogen donor ligands, namely "$An(S_2CNEt_2)_2(MeCN)_x$." Given the limited number of DFT studies involving minor actinides, our initial effort focused on establishing confidence in our computational approach through comparison of optimized versus observed $M(S_2CNEt_2)_2(N_2C_{12}H_8)$ structures. In this case, there was exceptional agreement between experiment and theory, as evident from the averaged bond distance comparisons in Table 1. Calculated $An–S_{(mean)}$ and $An–N_{N2C12H8(mean)}$ distances were only 0.02 and 0.06 Å different than analogous experimental values. Similarly good agreement was observed for the analogous distances in the Eu^{III} and Gd^{III} compounds.[11, 12] Emboldened by these results,

the experimentally isolated $M(S_2CNEt_2)_3(N_2C_{12}H_8)$ structure was computationally compared with the proposed acetonitrile adduct, "$M(S_2CNEt_2)_3(MeCN)_2$," which has yet to be characterized structurally. Average An–S and An–N bond distances for the MeCN adducts were calculated to be similar to those for the $M(S_2CNEt_2)_2(N_2C_{12}H_8)$ molecules, within 0.014 Å, respectively.

Thermodynamic metrics associated with Eq. 2 were computationally evaluated to directly probe the *f*-element's binding preference of $N_2C_{12}H_8$ over MeCN.

$$M(S_2CNEt_2)_3(N_2C_{12}H_8) + 2\ MeCN \longrightarrow M(S_2CNEt_2)_3(MeCN)_2 + N_2C_{12}H_8 \qquad \textbf{(2)}$$

M = Am, Cm, Cf, Eu, Gd

For all three actinides (Am, Cm, Cf) and two lanthanides (Ln = Eu, Gd), calculated reaction enthalpies and Gibbs free energies were similar (ranging 9.0 and 13.2 kcal/mol) and positive (Table 2). These results suggested that replacing $N_2C_{12}H_8$ by two MeCN was endergonic, and not thermodynamically favorable, which was consistent with experimental observations that $M(S_2CNEt_2)_3(N_2C_{12}H_8)$ crystallized as the sole product from MeCN solutions. Three variables that contribute to stabilization of $M(S_2CNEt_2)_3(N_2C_{12}H_8)$ and $An(S_2CNEt_2)_3(MeCN)_2$ were compared in Table 3, specifically electrostatic stabilization (the electron attraction term), Pauli repulsion (electron repulsion term), and orbital interactions (stabilization from covalency). Formation of both the $N_2C_{12}H_8$ and MeCN adducts were favorable in terms of electrostatic stabilization; the $M(S_2CNEt_2)_3N_2C_{12}H_8$ by *ca.* -74 kcal/mol and $M(S_2CNEt_2)_3(MeCN)_2$ by *ca.* -50 kcal/mol. However, this electrostatic stabilization was offset by Pauli repulsion energies, which were approximately 77 kcal/mol for $M(S_2CNEt_2)_3(N_2C_{12}H_8)$ and ~53 kcal/mol for $M(S_2CNEt_2)_3(MeCN)_2$. Hence, summing these two values (electron attraction and repulsion) resulted in small positive values (ranging 1.4 to 8.5 kcal/mol) for Am^{III}, Cm^{III}, Cf^{III}, and Gd^{III}. Results from Eu^{III} were similar; however, the absolute values of

the attraction and repulsion terms were slightly negative (-0.59 and -1.53 kcal/mol). Considering only these two metrics, one would predict that the $M(S_2CNEt_2)_3L_x$ complexes would not be stable for either $N_2C_{12}H_8$ or MeCN. However, large negative stabilization energies were obtained once the orbital interaction terms were considered. Moreover, these results indicated that the total bonding energies between fragments of $M(S_2CNEt_2)_3$ and $N_2C_{12}H_8$ were more negative than that between $M(S_2CNEt_2)_3$ and two MeCN ligands (difference of 5.9 to 8.2 kcal/mol). Hence these calculations suggested that the formation of $M(S_2CNEt_2)_3N_2C_{12}H_8$ was substantially more stable than the hypothetical MeCN adduct.

To obtain a better understanding of the An–L interactions in $An(S_2CNEt_2)_3L_x$ (An = Am, Cm, Cf; L = $N_2C_{12}H_8$, x = 1; MeCN, x = 2) , we computationally interrogated the M–N bonding interactions in the f^7 $Cm(S_2CNEt_2)_3L_x$ and $Gd(S_2CNEt_2)_3L_x$ compounds using natural orbitals for chemical valence (NOCV) analyses. Because the Gd^{III} and Cm^{III} theoretical results were nearly identical (see Figures S4 and S5) we will only discuss here the $Cm(S_2CNEt_2)_3L_x$ data. The NOCV deformation densities indicated that the Cm–L interaction for both $N_2C_{12}H_8$ and MeCN were dominated by σ-bonds. They also provided no appreciable evidence of π-backdonation from the metal occupied *f*-orbitals to ligand unoccupied orbitals. These primarily σ-bonding interactions consisted of N 2*p*-orbitals mixing with Cm^{III} 5*f*-, 6*d*-, and 7*s*-orbitals. The calculations also indicated that the $Cm–N_{N2C12H8}$ σ–interaction was slightly stronger than the analogous interactions with MeCN, consistent with stronger orbital interactions (Table 3). Overall, the computational results suggested that $N_2C_{12}H_8$ and MeCN were best described as σ-bonding ligands with minor actinides. Even the polycyclic aromatic $N_2C_{12}H_8$ ligand showed no potential for forming π–bonds with the minor actinides.

Outlook

Provided herein is evidence that the $M(S_2CEt_2)_3$(ligand) molecular scaffolding is compatible with Ln^{III}, An^{III}, and particularly +3 transplutonium elements. We identified that the $M(S_2CEt_2)_3$(ligand) compounds have two critical parameters that make them ideal for advancing understanding of how physical and chemical properties change across the *f*-element series. The $S_2CNEt_2^{1-}$ ligand framework provides air and moisture stability and facilitates formation of highly crystalline products. Moreover, the $M(S_2CNEt_2)_3$ fragment is sterically unsaturated and readily accommodates additional ligands. Note, with lanthanides, the $M(S_2CNEt_2)_3$ fragment provides access to diverse coordination chemistry.[30-42] Extending $M(S_2CNEt_2)_3$ coordination chemistry to include Am^{III}, Cm^{III}, and Cf^{III} provided a unique opportunity to evaluate M–N interactions in known $M(S_2CNEt_2)_3(N_2C_{12}H_8)$ versus proposed "$M(S_2CNEt_2)_3(MeCN)_2$" compounds. The results revealed that more pronounced $M–N_2C_{12}H_8$ covalent σ-bonding interactions (not π) directed formation of $An(S_2CNEt_2)_3(N_2C_{12}H_8)$ over the "$An(S_2CNEt_2)_3(Me_3CN)$" alternative, which was facilitated by delocalization of charge throughout the polycyclic aromatic backbone. If synthetic challenges associated with handling ^{243}Am, ^{248}Cm, and ^{249}Cf can continue to be circumvented, the foregoing results divulge a general method to potentially interrogate a series of actinide versus lanthanide–ligand interaction within a ubiquitous $M(S_2CEt_2)_3$(ligand) platform. The implications of such breakthroughs could have impact on defining how physical and chemical properties vary with periodicity across the *f*-element series, to the minor actinides (Am and Cm) and beyond (Cf).

Experimental Procedures

General Considerations. The 1,10-phenanthroline ($N_2C_{12}H_8$; Sigma Aldrich), sodium diethyldithiocarbamate (NaS_2CNEt_2; Sigma Aldrich), diethylammonium diethyldithiocarbamate, [$(NH_2Et_2)S_2CNEt_2$; Sigma Aldrich], acetonitrile (MeCN; Sigma Aldrich), anhydrous acetonitrile (MeCN; Sigma Aldrich), hydrochloric acid (HCl; 12 M, Fisher), hydrofluoric acid (HF; 28 M, Fisher), boric acid

(H_3BO_3; Fisher), ammonium hydroxide (NH_4OH; 14.5 M, Sigma Aldrich), hydrogen peroxide (H_2O_2, 30%, Fisher), Paratone-N oil (Hampton Research), and d_3-chloroform ($CDCl_3$; Sigma Aldrich) were obtained commercially and used as received. All water used in these experiments was deionized and passed through a Barnstead water purification system until a resistivity of 18 MΩ was achieved.

Caution! Because of spontaneous fission and emission of α-, β-, and γ-particles from ^{248}Cm [$t_{½}$ = 3.48(6)×10^5 years], ^{243}Am [$t_{1/2}$=7,364(22) years], and ^{249}Cf [$t_{1/2}$=351(2) years],[43] as well as their corresponding daughter products. The radioactive samples used herein represent serious health threats. Hence, all studies with ^{243}Am, ^{248}Cm, and ^{249}Cf were conducted in a radiation laboratory equipped with HEPA filtered hoods, continuous air monitors, and negative pressure gloveboxes. All free-flowing solids were handled within negative pressure gloveboxes whose exhaust ports were equipped with HEPA filters.

Single Crystal X-ray Diffraction. Single crystals of $Am(S_2CNEt_2)_3(N_2C_{12}H_8)$ and $Cf(S_2CNEt_2)_3(N_2C_{12}H_8)$ were mounted on a Mitogen mounts with krytox oil and the crystals were optically aligned on a Bruker D8 Quest X-ray diffractometer using a built-in camera. Preliminary measurements were performed using an Ims X-ray source (Mo Ka, λ = 0.71073 A) with high-brilliance and high-performance focusing quest multilayer optics. APEX II software was used for solving the unit cells and data collection. The reflection's intensities of a sphere were collected by a combination of four sets of frames. Each set had a different omega angle for the crystal, and each exposure covered a range of 0.50 in ω totaling to 1464 frames. The frames were collected with an exposure time of 5-25 seconds, which was dependent on the crystal. SAINT software was used for data integration including polarization and Lorentz corrections. PLATON was used to check the structure for missed symmetry and twinning.[44]

Single crystals of $Cm(S_2CNEt_2)_3(N_2C_{12}H_8)$ were mounted with three appropriate layers of containment prior to single crystal X-ray diffraction studies.[45] The diffraction study of Cm was performed on a D8 Bruker QUEST diffractometer with a sealed tube Mo source (Mo Ka, λ = 0.71073 A). No corrections for crystal decay were necessary. Standard APEX II software was used for determination of the unit cells and data collection control. The intensities of the reflections of a sphere were collected by combining four sets of exposures (frames), which totaled to 1464 frames with an exposure time of 5 seconds per frame. APEX II software was used for data integration including Lorentz and polarization corrections. The crystal structure was solved using SHELX software and PLATON was used to check the Crystallographic Information Files (CIFs) for missed symmetry and twinning.[44] The CIF files used in this manuscript are available through the Cambridge Crystal Data Centre (CCDC; 1841778, 1841779, 1841780).

UV-vis-NIR and Fluorescence. Single crystals were placed on a quartz slide under oil and spectra obtained using a Craic Technologies microspectrophotometer. For absorbance measurements, data was collected from 40,000 to 9,090.91 cm^{-1} (250 to 1100 nm). Meanwhile, fluorescence spectra were obtained with an excitation at 23,809.52 cm^{-1} (420 nm) or 27,396.26 cm^{-1} (365 nm).

FT-IR. Single crystals were placed on the iD7 ATR crystal window (for An samples this was performed inside of a negative pressure glove box), and transmission spectra were collected from 410 to 3300 cm^{-1} on a Nicolet™ iS™ 5N FT-NIR.

Preparation of Cm^{III} Stock Solution. In a HEPA filtered open front fume hood and under an atmosphere of air, a Cm^{III} stock solution was prepared as shown in Scheme 1. Residues known to contain ^{248}Cm that had been used in previous experimental campaigns were dissolved in hydrochloric acid (HCl, 6 M). The

dissolved samples were combined in a beaker and gently heated. After the aqueous solvent was evaporated to a soft dryness under a stream of air the residue was dissolved in HCl (6 M) and transferred into a single falcon tube. Hydrofluoric acid (HF, 28 M, 5 mL) was added to this solution resulting in a faint yellow precipitate. After 15 minutes, the suspension was centrifuged and the supernatant (clear) was removed leaving behind a pale yellow pellet. The pellet was washed with water (2x) and dissolved by using a combination of heating and the addition of H_3BO_3 (2 mL), while agitating the pellet with a glass stir rod. After heating (80 °C; 5 min), HCl (12 M, 1 mL) was added and the mixture heated for another 5 minutes. While maintaining the temperature near 80 °C, aliquots of H_3BO_3 (1 mL) and HCl (1 mL) were added sequentially with periodic stirring. Upon complete dissolution of the solid, ammonium hydroxide (NH_4OH, 14.5 M) was added drop wise while stirring until a solid precipitated, naively formulated as $Cm(OH)_3$. The suspension was centrifuged, the supernatant discarded, and the remaining pellet was washed with water (2x). The solid pellet was then dissolved in HCl (12 M, 6 drops) and diluted with water (10 mL), which generated a slightly acidic solution containing Cm^{III}.

A cation-exchange column was prepared by charging a BioRad column (10 mL) with AG 50 x 8 cation resin (2 mL, 100 – 200 mesh). The resin was first conditioned with HCl (12 M; 4 x 5 mL), then with dilute HCl (0.1 M; 4 x 5 mL), and lastly with water (4 x 5 mL). The Cm^{III} solution, which was in dilute acid, was loaded onto the column. Under these conditions, Cm^{III} was retained on the resin. The column was washed with HCl (0.1 M; 3 x 10 mL), then Cm^{III} was eluted with concentrated HCl (12 M; 5 x 10 mL). The Cm^{III} elution profile was quantified by stippling small volumes (one drop) of the eluted fraction onto Pyrex slides. After the samples dried under air, gross ^{248}Cm α-activity was quantified by analyzing each slide using a Ludlum 3939E α-, β-, γ-stationary survey instrument (for low activity samples) or a hand held portable α-survey meter (Ludlum 139) for high activity samples. Afterwards, stippled ^{248}Cm was recovered by soaking the slides in HCl (6 M) and set aside to be reprocessed at a later date. The five Cm^{III}

elution fractions were combined and the solution was heated to a soft dryness. The resulting residue was dissolved in HCl (6 M; 5 mL), giving a chemically and radiochemically pure Cm^{III} stock solution. The Cm^{III} concentration was determined by analyzing an aliquot (100 μL) of the stock solution in HCl (2 M, 2.5 mL) by UV-vis spectroscopy. For these measurements we assumed the 25,220.68 cm^{-1} (396.5 nm) absorbance had an extinction coefficient of 52.9 $Lmol^{-1}cm^{-1}$, as previously reported.[27,46]

Preparation of Cf^{III} Stock Solution. In a HEPA filtered open front fume hood a Cf^{III} stock solution was prepared. Residues known to contain ^{249}Cf that had been used in previous experimental campaigns were dissolved in hydrochloric acid (HCl, 6 M). The ^{249}Cf samples were combined in a beaker and gently heated. After the aqueous solvent evaporated under a stream of air (a soft dryness) the residue was dissolved in HCl (6 M) and transferred into a single falcon tube.

Due to possible iron contaminates in the ^{249}Cf sample, an anion-exchange column was prepared by charging a BioRad column (20 mL) with AG MP-1 (5 mL; 50-100 mesh). The resin was conditioned with water (3 x 10 mL), HCl (12 M; 3 x 10 mL), followed by an additional HCl wash (12 M; 30 mL). The ^{249}Cf was loaded onto the top of column and washed with HCl (12 M; 10 x 5 mL). All fractions were analyzed by γ-spectroscopy to determine when the ^{249}Cf was completely eluted off of the column. The ^{249}Cf was collected and the volume was reduced to 13 mL. Water was then added to the sample to give a 1 M HCl ^{249}Cf stock solution.

A cation-exchange column was prepared by charging a BioRad column (20 mL) with AG 50 x 8 cation resin (3 mL, 100 – 200 mesh). The resin was conditioned with water (3 x 10 mL), followed by HCl (6 M; 3 x 10 mL), and finally with dilute HCl (1 M; 3 x 10 mL). The Cf^{III} solution was loaded onto the column in dilute HCl (1 M). Under these conditions, Cf^{III} was retained on the resin. The column was washed with HCl (0.1 M; 3 x 10 mL). Elution of Cf^{III} was achieved with concentrated HCl (12 M; 3 x 10 mL), which

was monitored using γ-spectroscopy by following the 333.37(2) KeV [15.0(6)% branching ratio] and 388.17(2) KeV [66.0(24) %] ^{249}Cf γ-emission.[1] The elution fraction were collected and combined. Then the solution was heated to a soft dryness under a heating lamp and a stream of filtered air. The resulting residue was dissolved in HCl (6 M; 5 mL) giving a chemically and radiochemically pure Cf^{III} stock solution. The Cf^{III} concentration was determined by analyzing an aliquot (100 μL) of the stock solution in HCl (2 M; 2.5 mL) by γ-spectroscopy.

Americium(III) tris-diethyl(dithiocarbamate) 1,10-phenanthroline, $Am(S_2CNEt_2)_3(N_2C_{12}H_8)$. In a HEPA filtered open front fume hood and under an atmosphere of air, AmO_2 (5.0 mg, 0.011 mmol) was dissolved in nitric acid (300 mL; 6 M) and the solution was evaporated to dryness. This was repeated twice to fully covert the AmO_2 to $Am(NO_3)_3{\cdot}X(H_2O)$. The Am residue (5.0 mg, 0.011 mmol) was dissolved in acetonitrile (MeCN; 0.5 mL) and a solution of diethylammonium dithiocarbamate $(NH_2Et_2)S_2CNEt_2$; 7.6 mg, 0.034 mmol) and 1,10-phenanthroline ($N_2C_{12}H_8$; 2.1 mg, 0.011 mmol) in anhydrous acetonitrile (0.5 mL) was added to the vial. Subsequently, the solution turned bright yellow. After 20 minutes yellow rod shaped crystals formed that were suitable for single crystal X-ray diffraction. UV-vis [cm^{-1}]: 24880.8, 23005.7, 22187.3, 22067.4, 21437.0, 20436.85, 19207.13 ($^5L_6$), 12231.5, 12311.16 ($^7F_5$), 11460.29, 11317.9, 11212.0, 10957.2.

Curium(III) tris-diethyl(dithiocarbamate) 1,10-phenanthroline, $Cm(S_2CNEt_2)_3(N_2C_{12}H_8)$. In a HEPA filtered open front fume hood and under an atmosphere of air, an aliquot of the ^{248}Cm stock solution (1.5 mL, 0.0135 M) in HCl (6 M) was added to a scintillation vial (20 mL). The solvent was removed (to a soft dryness) by heating the flask until a pale yellow residue formed. The solution matrix was converted to nitric acid by dissolving the residue in HNO_3 (8 M, 1 mL), heating the vial to remove the aqueous solution,

and dissolving the resulting residue in HNO_3 (8 M, 1 mL) again. After repeating this procedure a total of three times, a residue was isolated that was assumed to be $Cm(NO_3)_3{\cdot}X(H_2O)$. The vial was slowly cooled to room temperature, acetonitrile (MeCN; 0.5 mL) was added, and the "$Cm(NO_3)_3{\cdot}X(H_2O)$" residue completely dissolved. The solution was transferred to a centrifuge tube (50 mL) and the vial washed with an additional aliquot of MeCN (0.5 mL). Subsequent addition of sodium diethyldithiocarbamate (NaS_2CNEt_2; 13.6 mg, 0.0606 mmol) caused a precipitate to form immediately. This precipitate was removed by centrifugation and the supernate transferred to a clean scintillation vial. At this point 1,10-phenanthroline ($N_2C_{12}H_8$; 4.2 mg, 0.023 mmol) was added to the vial. Slow evaporation of the solvent for 14 hours enabled pale yellow/orange crystals suitable for characterization by single crystal X-ray diffraction to form. UV-vis [cm^{-1}]: 16,433.2 cm^{-1} ($^6P_{7/2}$), 16,537.6 cm^{-1} ($^6P_{7/2}$), 16,806.97 cm^{-1} ($^6D_{7/2}$), 19,732.3 cm^{-1} ($^6P_{5/2}$), 19,802.75 cm^{-1} ($^6P_{5/2}$), 21,376.66 cm^{-1} ($^6I_{7/2}$), 21,927.06 cm^{-1} ($^6P_{3/2}$), 22,517.48 cm^{-1} ($^6I_{9/2}$). Fluorescence [cm^{-1}]: 16368.33. IR [cm^{-1}]: 1458.45 s, 1418.78 s, 1284.12 s, 1030.93 s, 848.69 s, 732.02 s, 720.18 s, 1371.68 m, 1204.39 m, 1141.74 m, 1103.82 m, 995.84 m, 863.05 m, 781.46 m, 634.16 m, 426.07 m, 1624.48 w, 1590.62 w, 1575.48 w, 1518.49 w, 1345.59 w, 910.54 w, 893.46 w, 810.49 w, 573.55 w, 501.98 w, 477.69 w, 451.28 w, 435.12 w.

Californium(III) tris-diethyl(dithiocarbamate) 1,10-phenanthroline, $Cf(S_2CNEt_2)_3(N_2C_{12}H_8)$. As described above for $Am(S_2CNEt_2)_3(N_2C_{12}H_8)$, the $Cf(S_2CNEt_2)_3(N_2C_{12}H_8)$ coordination complex was prepared by adding $Cf(NO_3)_3{\cdot}X(H_2O)$ (5.0 mg, 0.012 mmol) to acetonitrile (0.5 mL), diethylammonium dithiocarbamate $(NH_2Et_2)SCNEt_2$; 7.7 mg, 0.040 mmol), and 1,10-phenanthroline ($N_2C_{12}H_8$; 2.1 mg, 0.012 mmol). Green rod-shaped crystals were obtained by slow evaporation of acetonitrile (0.5 mL) within 20 minutes that were suitable for single crystal X-ray diffraction. UV-vis [cm^{-1}]: 11,100.7 cm^{-1} ($^6H_{11/2}$),

11,149.4 cm^{-1} ($^6H_{11/2}$), 11,322.6 cm^{-1} ($^6H_{11/2}$), 11,448.3 cm^{-1} ($^6H_{11/2}$), 11,492.1 cm^{-1} ($^6H_{11/2}$), 11,641.8 cm^{-1} ($^6H_{11/2}$), 11,686.5 cm^{-1} ($^6H_{11/2}$), 11,727.25 cm^{-1} ($^6H_{11/2}$), 12,792.03 cm^{-1} ($^6H_{9/2}$), 13,134.9 cm^{-1} ($^6F_{7/2}$), 14,394.8 cm^{-1} ($^6H_{7/2}$), 14,426.5 cm^{-1} ($^6H_{7/2}$), 16,104.6 cm^{-1} ($^6H_{5/2}$), 16,125.01 cm^{-1} ($^6F_{5/2}$), 20,174.7 cm^{-1} ($^6P_{5/2}$), 20,225.9 cm^{-1} ($^4K_{17/2}$), 21,108.7 cm^{-1} ($^4K_{17/2}$), 22,313.7 cm^{-1} ($^6\mathrm{F}_{3/2}$, $^6\mathrm{P}_{7/2}$, $^4\mathrm{L}_{19/2}$, $^4\mathrm{M}_{21/2}$), 22,301.9 cm^{-1} ($^6\mathrm{F}_{3/2}$, $^6\mathrm{P}_{7/2}$, $^4\mathrm{L}_{19/2}$, $^4\mathrm{M}_{21/2}$), 22,948.6 cm^{-1} ($^2I_{13/2}$).Raman [cm^{-1}]: 45707, 24483, 16138, 13250, 12624, 11858, 11070, 9996.8, 9722, 9133.8, 8683.6, 8464.2, 8448.4, 6918.2, 6323, 6244.8.

Density Functional Calculations. Ground-state electronic structure calculations were performed on the $M(S_2CNEt_2)_3(N_2C_{12}H_8)$ and $M(S_2CNEt_2)_3$(MeCN) complexes and $N_2C_{12}H_8$ and MeCN ligands using the hybrid functional PBE0[17] implemented in the Gaussian 09.[47] All the geometries were optimized in the gas phase. Harmonic frequency calculations were performed to obtain the corresponding thermochemical corrections and to confirm that the optimized structures were stationary points on the potential energy surface. For each molecule, a subsequent single point using the implicit CPCM solvation model[48] with the Universal Force Field (UFF) radii[49] and Solvent Excluding Surface (SES)[50,51] was calculated to account for the solvation effects. A relative permittivity of 35.688 was assumed in the solvation calculations to simulate acetonitrile as the solvent experimentally used for all the cases. We applied the 6-311G* basis sets[52,53] for non-metal atoms, Stuttgart energy-consistent relativistic pseudopotentials ECP28MWB[54,55] and the corresponding ECP28MWB-SEG basis for lanthanide atoms, and Stuttgart energy-consistent relativistic pseudopotentials ECP60MWB[56,57] and the corresponding ECP60MWB-SEG basis for actinide atoms.

To interrogate metal binding of $N_2C_{12}H_8$ ligand over MeCN, further bonding analyses were performed with the energy decomposition approach (EDA).[58-60] To evaluate the relative importance of steric repulsion and orbital interactions between $M(S_2CNEt_2)_3$ fragment and ligand fragment in the $M(S_2CNEt_2)_3$(Ligand)

(Ligand=$N_2C_{12}H_8$ or $(MeCN)_2$) compounds, and the M-N σ and/or π bonding components in the orbital interaction was further analyzed using an extended transition state energy decomposition scheme combined with the natural orbitals for chemical valence (ETS-NOCV).[61] The calculations were performed at the above optimized geometries using PBE0 functional, scalar relativistic ZORA Hamiltonian[62], and triple-ζ plus two polarization functions (TZ2P)[63] basis sets with the frozen core approximation applied to the inner shells [$1s^2$-$4f^{14}$] for actinide atoms and [$1s^2$-$4d^{10}$] for lanthanides and [$1s^2$] for C and N and [$1s^2$-$2p^6$] for S implemented in the Amsterdam Density Functional (ADF 2016.104)[43, 64, 65].

Supporting Information.

All experimental (including IR, Raman, cryptographic tables) and computational (NOCV analysis results, optimized Cartesian coordinates) compounds can be found in the supporting information.

Acknowledgement

We gratefully recognize the Heavy Element Chemistry Program at LANL by the Division of Chemical Sciences, Geosciences, and Biosciences, Office of Basic Energy Sciences, U.S. Department of Energy and the U.S. Department of Energy for funding the synthesis and characterization of the $Cm(S_2CNEt_2)_3(N_2C_{12}H_8)$ complex. Los Alamos National Laboratory is operated by Los Alamos National Security, LLC, for the National Nuclear Security Administration of U.S. Department of Energy (contract DE-AC52-06NA25396). We additionally thank the U.S. Department of Energy, Office of Science, Office of Basic Energy Sciences, Heavy Elements Chemistry Program, under Award Numbers DE-FG02-13ER16414 for the synthesis and characterization of $Cf(S_2CNEt_2)_3(N_2C_{12}H_8)$ (Albrecht-Schmitt, Galley, Alstine). The synthesis of $Am(S_2CNEt_2)_3(N_2C_{12}H_8)$, the computational work for the $M(S_2CNEt_2)_3$(ligand) compounds, and the Cm and Cf reprocessing efforts were supported as part of the Center for Actinide

Science and Technology (CAST) an Energy Frontier Research Center (EFRC) funded by the U.S. Department of Energy (DOE), Office of Science, Basic Energy Sciences (BES), under Award Number DE-SC0016568. In addition, portions of this work were supported by postdoctoral fellowships from the Glenn T. Seaborg Institute (Ferrier, Su) and the LDRD office, named fellowship program; Hoffman Distinguished Postdoctoral Fellowship (Cary).

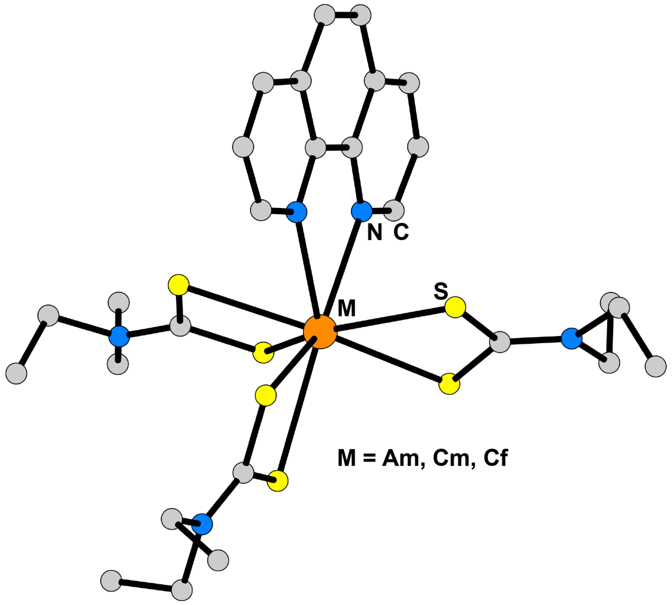

Figure 1. A ball and stick representation of the $An(S_2CNEt_2)_3(N_2C_{12}H_8)$ crystal structures (An = Am, Cm, Cf).

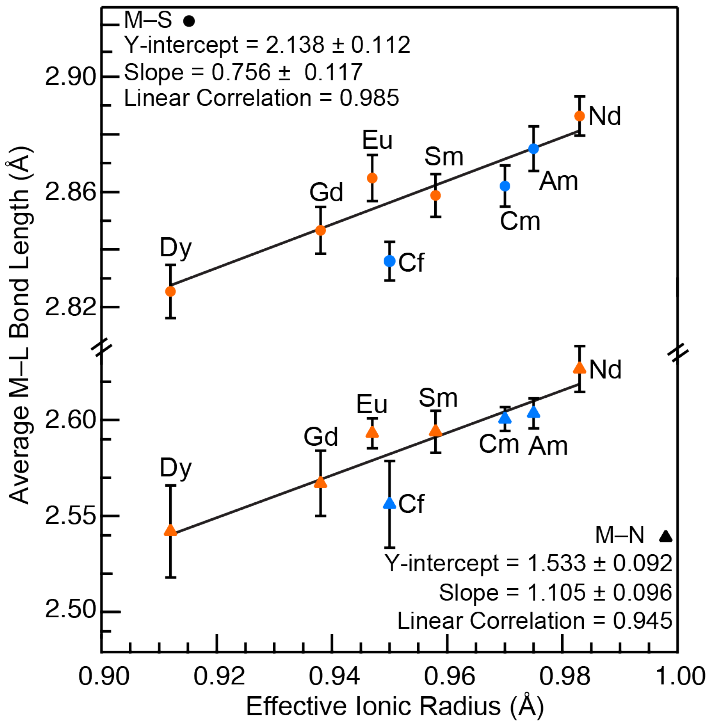

Figure 2. Comparison of the mean M–S (●; *top*) and M–N (▲; *bottom*) bond lengths in $M(S_2CNEt_2)_3(N_2C_{12}H_8)$ (M = Nd, Sm, Eu, Gd, Dy, Am, Cm, and Cf) versus six-coordinate metal ionic radii.[11, 12, 16] Lanthanides are represented by orange symbols and actinides by blue symbols. Uncertainty is reported as standard deviation from the mean (1σ). Data were fit with a line ($y = mx + b$), as shown by the solid black trace.

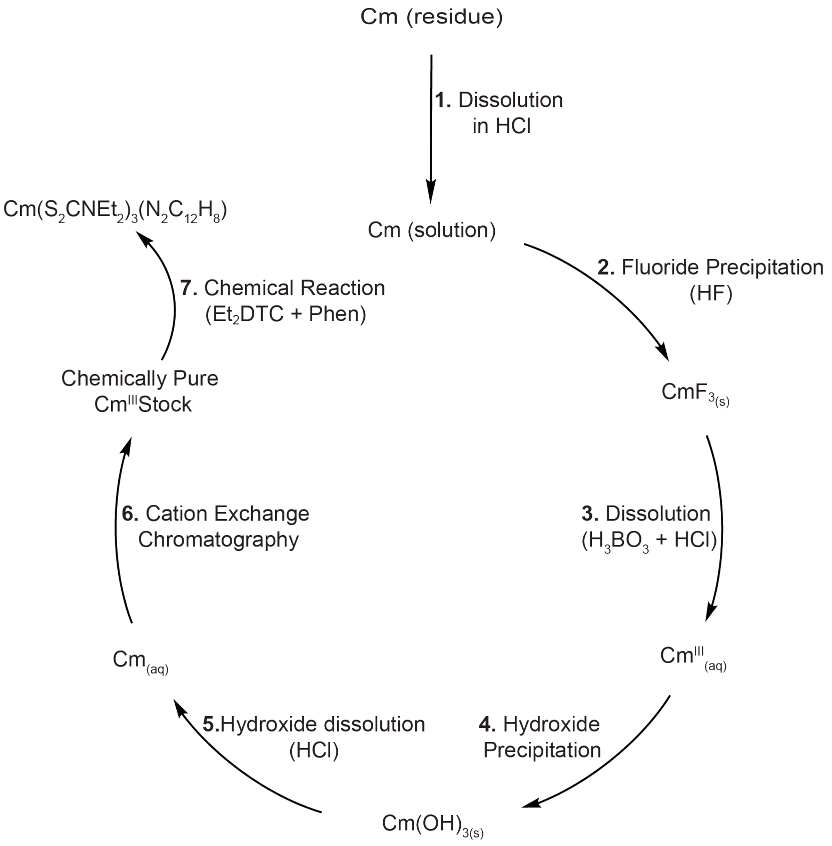

Scheme 1. A schematic representing the Cm recovery and reprocessing strategy.

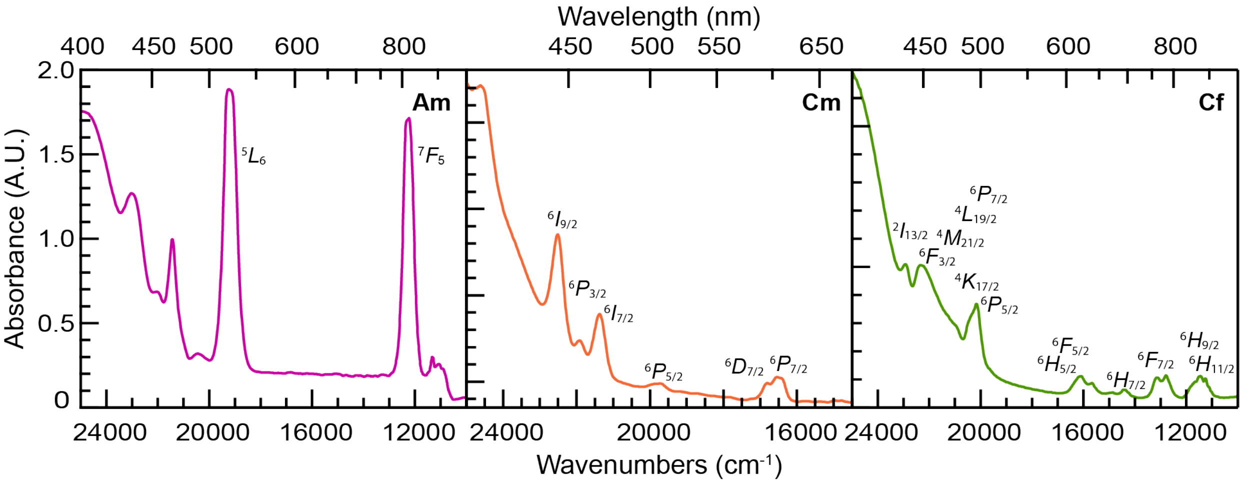

***Figure 3*.** Solid state UV-vis-NIR absorbance of $An(S_2CNEt_2)_3(N_2C_{12}H_8)$ (An = Am, Cm, Cf; pink, orange, and green traces).

Table 1. The optimized average M–S and M–N bond lengths (Å) calculated for $M(S_2CNEt_2)_3(N_2C_{12}H_8)$ and $M(S_2CNEt_2)_2(MeCN)_2$ (M = Am, Cm, Cf, Eu, Gd) compared with experimental data, when available.

	$M(S_2CNEt_2)_3(N_2C_{12}H_8)$				$M(S_2CNEt_2)_3(MeCN)_2$	
M	Average M–S		Average M–N		M–S	M–N
	Theory	Expt.	Theory	Expt.	Theory	Theory
Am	2.8802	2.8745 ± 0.05	2.6621	2.6031 ± 0.008	2.8700	2.6498
Cm	2.8782	2.8620 ± 0.04	2.6450	2.6009 ± 0.006	2.8684	2.6377
Cf	2.8409	2.8355 ± 0.04	2.6099	2.5562 ± 0.023	2.8310	2.5962
Eu	2.8681	2.8547 ± 0.05	2.6387	2.5858 ± 0.013	2.8619	2.6217
Gd	2.8441	2.8467 ± 0.05	2.6021	2.5670 ± 0.017	2.8318	2.5984

Table 2. Enthalpy and Gibbs free energy (in kcal/mol) for the substitution reaction of $N_2C_{12}H_8$ for MeCN: $M(S_2CNEt_2)_3(N_2C_{12}H_8)$ + 2 MeCN $\rightarrow$ $M(S_2CNEt_2)_3(MeCN)_2$ + $N_2C_{12}H_8$ in MeCN solution.

M	Am	Cm	Cf	Eu	Gd
ΔH_{solv}	9.8	10.1	9.2	9.0	10.1
ΔG_{solv}	13.1	12.8	12.6	12.6	13.2

Table 3. Energy decomposition analysis of the total bonding interaction between fragments $M(S_2CNEt_2)_3$ and ligands in $M(S_2CNEt_2)_3$(Ligand) (M = Eu, Gd, Am, Cm, and Cf; Ligand = $N_2C_{12}H_8$ or MeCN)$_2$) reported in kcal/mol.

M	Steric Interactions			Orbital Interactions	Total Bonding Energy[b]
	Electrostatic Interactions	Pauli Repulsion	Sum[a]		
			$M(S_2CNEt_2)_3(N_2C_{12}H_8)$		
Am	-75.40	78.61	3.20	-58.09	-54.89
Cm	-75.23	79.17	3.94	-71.29	-67.36
Cf	-76.03	83.53	7.50	-67.64	-60.14
Eu	-70.17	68.63	-1.53	-66.54	-68.07
Gd	-74.88	76.27	1.39	-79.03	-77.65
			$M(S_2CNEt_2)_3(MeCN)_2$		
Am	-50.85	54.95	4.10	-52.19	-48.09
Cm	-49.77	54.19	4.42	-64.20	-59.77
Cf	-50.76	59.28	8.52	-60.95	-52.43
Eu	-48.01	47.42	-0.59	-59.44	-60.03
Gd	-49.19	50.64	1.45	-70.83	-69.38

[a] Steric interaction is the sum of electrostatic and Pauli interactions.

[b] Total bonding energy is the sum of steric interactions and orbital interactions.

REFERENCES.

1. G. R. Choppin, *J. Less-Common Met.*, 1983, **93**, 323-330.
2. M. Nilsson and K. L. Nash, *Solvent Extr. Ion Exch.* , 2007, **25**, 665-701.
3. S. K. Cary, M. Vasiliu, R. E. Baumbach, J. T. Stritzinger, T. D. Green, K. Diefenbach, J. N. Cross, K. L. Knappenberger, G. Liu, M. A. Silver, A. E. DePrince, M. J. Polinski, S. M. Van Cleve, J. H. House, N. Kikugawa, A. Gallagher, A. A. Arico, D. A. Dixon and T. E. Albrecht-Schmitt, *Nat. Commun.*, 2015, **6**, 6827.
4. M. J. Polinski, E. B. Garner, 3rd, R. Maurice, N. Planas, J. T. Stritzinger, T. G. Parker, J. N. Cross, T. D. Green, E. V. Alekseev, S. M. Van Cleve, W. Depmeier, L. Gagliardi, M. Shatruk, K. L. Knappenberger, G. Liu, S. Skanthakumar, L. Soderholm, D. A. Dixon and T. E. Albrecht-Schmitt, *Nat. Chem.*, 2014, **6**, 387-392.
5. S. K. Cary, M. A. Silver, G. Liu, J. C. Wang, J. A. Bogart, J. T. Stritzinger, A. A. Arico, K. Hanson, E. J. Schelter and T. E. Albrecht-Schmitt, *Inorg. Chem.*, 2015, **54**, 11399-11404.
6. M. A. Silver, S. K. Cary, J. A. Johnson, R. E. Baumbach, A. A. Arico, M. Luckey, M. Urban, J. C. Wang, M. J. Polinski, A. Chemey, G. Liu, K. W. Chen, S. M. Van Cleve, M. L. Marsh, T. M. Eaton, L. J. van de Burgt, A. L. Gray, D. E. Hobart, K. Hanson, L. Maron, F. Gendron, J. Autschbach, M. Speldrich, P. Kogerler, P. Yang, J. Braley and T. E. Albrecht-Schmitt, *Science*, 2016, **353**, 888.
7. S. K. Cary, K. S. Boland, J. N. Cross, S. A. Kozimor and B. L. Scott, *Polyhedron*, 2017, **126**, 220-226.
8. J. N. Cross, J. A. Macor, J. A. Bertke, M. G. Ferrier, G. S. Girolami, S. A. Kozimor, J. R. Maassen, B. L. Scott, D. K. Shuh, B. W. Stein and S. C. E. Stieber, *Angew. Chem. Int. Ed.*, 2016, **55**, 12755-12759.
9. S. K. Cary, M. Livshits, J. N. Cross, M. G. Ferrier, V. Mocko, B. W. Stein, S. A. Kozimor, B. L. Scott and J. J. Rack, *Inorg. Chem.*, 2018, **57**, 3782–3797.
10. M. J. Polinski, S. Wang, E. V. Alekseev, W. Depmeier and T. E. Albrecht-Schmitt, *Angew Chem. Int. Ed Engl.*, 2011, **50**, 8891-8894.
11. M. D. Regulacio, M. H. Pablico, J. A. Vasquez, P. N. Myers, S. Gentry, M. Prushan, S.-W. Tam-Chang and S. L. Stoll, *Inorg. Chem.*, 2008, **47**, 1512-1523.
12. M. D. Regulacio, N. Tomson and S. L. Stoll, *Chem. Mater*, 2005, **17**, 3114-3121.
13. A. E. V. Gorden, J. Xu and K. N. Raymond, *Chem. Rev.* , 2003, **103**, 4207-4282.
14. J. Xu, E. Radkov, M. Ziegler and K. N. Raymond, *Inorg. Chem.*, 2000, **39**, 4156-4164.
15. J. K. Burdett, R. Hoffmann and R. C. Fay, *Inorg. Chem.*, 1978, **17**, 2553-2568.
16. R. D. Shannon, *Acta Cryst.*, 1976, **A32**, 751-767.
17. R. Pappalardo, W. T. Carnall and P. R. Fields, *J. Chem. Phys*, 1969, **51**, 842-843.
18. J. N. Cross, E. M. Villa, S. Wang, J. Diwu, M. J. Polinski and T. E. Albrecht-Schmitt, *Inorg. Chem.*, 2012, **51**, 8419-8424.
19. W. T. Carnall, P. R. Fields, D. C. Stewart and T. K. Keenan, *J. Inorg. Nucl. Chem.*, 1958, **6**, 213-216.
20. W. T. Carnall, S. Fried and F. Wagner, *J. Chem. Phys*, 1973, **58**, 1938-1949.
21. W. A. Runciman, *J. Chem. Phys*, 1962, **36**, 1481-1489.
22. B. G. Wybourne, *J. Chem. Phys*, 1964, **40**, 1456-1457.
23. J. B. Gruber, W. R. Cochran, J. G. Conway and A. T. Nicol, *J. Chem. Phys*, 1966, **45**, 1423-1427.

24. M. J. Polinski, S. Wang, E. V. Alekseev, W. Depmeier, G. Liu, R. G. Haire and T. E. Albrecht-Schmitt, *Angew Chem. Int. Ed Engl.*, 2012, **51**, 1869-1872.
25. M. J. Polinski, K. A. Pace, J. T. Stritzinger, J. Lin, J. N. Cross, S. K. Cary, S. M. Van Cleve, E. V. Alekseev and T. E. Albrecht-Schmitt, *Chemistry*, 2014, **20**, 9892-9896.
26. W. T. Carnall, J. V. Beitz and H. Crosswhite, *J. Chem. Phys*, 1984, **80**, 2301-2308.
27. D. L. Clark, S. S. Hecker, G. D. Jarvinen and M. P. Neu, *The Chemistry of the Actinide and Transactinide Elements*, Springer, Berlin, Germany, 2006.
28. G. Liu, S. K. Cary and T. E. Albrecht-Schmitt, *Phys. Chem. Chem. Phys.*, 2015, **17**, 16151-16157.
29. J. I. Kim, R. Klenze and H. Wimmer, *Eur. J. Solid State Inorg. Chem.*, 1991, **28**, 347-356.
30. I. Baba, I. Raya, B. M. Yamin and S. W. Ng, *Acta Crystallogr Sect E Struct Rep Online*, 2009, **65**, m1376.
31. S. Chengyong, T. Ning, T. Minyu and Y. Kaibei, *Polyhedron*, 1996, **15**, 233-239.
32. J. F. Bower, S. A. Cotton, J. Fawcett, R. S. Hughes and D. R. Russell, *Polyhedron*, 2003, **22**, 347-354.
33. V. Kubat, G. Demo, L. Jeremias and J. Novosad, *Z. Kristallogr. - New Cryst. Struct*, 2013, **228**, 369-373.
34. S. S. Liu, K. Lang, Y. Q. Zhang, Q. Yang, B. W. Wang and S. Gao, *Dalton Trans.*, 2016, **45**, 8149-8153.
35. M. Mahato, P. P. Jana, K. Harms and H. P. Nayek, *RSC Adv.*, 2015, **5**, 62167-62172.
36. P. B. Hitchcock, A. G. Hulkes, M. F. Lappert and Z. Li, *Dalton Trans.*, 2004, DOI: 10.1039/, 129-136.
37. P. Pitchaimani, K. M. Lo and K. P. Elango, *Polyhedron*, 2013, **54**, 60-66.
38. P. Pitchaimani, K. M. Lo and K. P. Elango, *J. Coord. Chem.*, 2015, **68**, 2167-2180.
39. P. Pitchaimani, K. M. Lo and K. P. Elango, *Polyhedron*, 2015, **93**, 8-16.
40. R. S. Selinsky, J. H. Han, E. A. Morales Pe´rez, I. A. Guzei and S. Jin, *J. Am. Chem. Soc*, 2010, **132**, 15997–16005.
41. C.-Y. Su, M.-Y. Tan, Z.-F. Zhang, N. Tang, L.-P. Cai and Q.-J. Xue, *Synth. React. Inorg. Metal-Org. nano-Met. Chem.*, 1999, **29**, 35-51.
42. H. Yin, P. J. Carroll and E. J. Schelter, *Chem. Commun.*, 2016, **52**, 9813-9816.
43. G. Te Velde, F. M. Bickelhaupt, E. J. Baerends, C. Fonseca Guerra, S. J. A. Van Gisbergen, J. G. Snijders and T. Ziegler, *Journal of Computational Chemistry*, 2001, **22**, 931-967.
44. A. L. Spek, *J. Appl. Cryst.*, 2003, **36**, 7-13.
45. S. K. Cary, K. S. Boland, J. N. Cross, S. A. Kozimor and B. L. Scott, *Polyhedron*, 2017, **126**, 220-226.
46. W. T. Carnall, P. R. Fields, D. C. Stewart and T. K. Keenan, *J. Inorg. Nucl. Chem*, 1958, **6**, 213-216.
47. C. Adamo and V. Barone, *J. Chem. Phys.*, 1999, **110**, 6158-6170.
48. M. J. Frisch, G. W. Trucks, H. B. Schlegel, G. E. Scuseria, M. A. Robb, J. R. Cheeseman, G. Scalmani, V. Barone, B. Mennucci, G. A. Petersson, H. Nakatsuji, M. Caricato, X. Li, H. P. Hratchian, A. F. Izmaylov, J. Bloino, G. Zheng, J. L. Sonnenberg, M. Hada, M. Ehara, K. Toyota, R. Fukuda, J. Hasegawa, M. Ishida, T. Nakajima, Y. Honda, O. Kitao, H. Nakai, T. Vreven, J. A. Montgomery, Jr., J. E. Peralta, F. Ogliaro, M. Bearpark, J. J. Heyd, E. Brothers, K. N. Kudin, V. N. Staroverov, R. Kobayashi, J. Normand, K. Raghavachari, A. Rendell, J. C. Burant, S. S. Iyengar, J. Tomasi, M. Cossi, N. Rega, J. M. Millam, M. Klene, J. E. Knox, J. B. Cross, V. Bakken, C. Adamo, J. Jaramillo, R. Gomperts, R. E. Stratmann, O. Yazyev, A. J.

Austin, R. Cammi, C. Pomelli, J. W. Ochterski, R. L. Martin, K. Morokuma, V. G. Zakrzewski, G. A. Voth, P. Salvador, J. J. Dannenberg, S. Dapprich, A. D. Daniels, Ö. Farkas, J. B. Foresman, J. V. Ortiz, J. Cioslowski and D. J. Fox, *Gaussian 09, revision D.01*, Gaussian Inc, 2013.

49. M. Cossi, N. Rega, G. Scalmani and V. Barone, *J. Comput. Chem.*, 2003, **24**, 669-681.
50. A. K. Rappe, C. J. Casewit, K. S. Colwell, W. A. I. Goddard and W. M. Skiff, *J. Am. Chem. Soc.*, 1992, **114**, 10024-10039.
51. M. L. Connolly, *J. Appl. Crystallogr.*, 1983, **16**, 548-558.
52. M. L. Connolly, *J. Mol. Graphics Modell.*, 1993, **11**, 139-141.
53. R. Krishnan, J. S. Binkley, R. Seeger and J. A. Pople, *J. Chem. Phys.*, 1980, **72**, 650.
54. A. D. McLean and G. S. Chandler, *J. Chem. Phys.*, 1980, **72**, 5639.
55. M. Dolg, H. Stoll and H. Preuss, *J. Phys. Chem.*, 1989, **90**, 1730-1734.
56. X. Cao and M. Dolg, *J. Mol. Struct.: THEOCHEM*, 2002, **581**, 139-147.
57. X. Cao, M. Dolg and H. Stoll, *J. Phys. Chem.*, 2003, **118**, 487-496.
58. X. Cao and M. Dolg, *J. Molec. Struct.*, 2004, **673**, 203-209.
59. T. Ziegler and A. Rauk, *Theor. Chim. Acta*, 1977, **46**, 1-10.
60. F. M. Bickelhaupt and E. J. Baerends, 2000, **15**, 1-86.
61. M. von Hopffgarten and G. Frenking, *Comput. Mol. Sci.*, 2012, **2**, 43-62.
62. M. Mitoraj, M. A and T. Ziegler, *J. Chem. Theory Comput.*, 2009, **5**, 962−975.
63. E. Van Lenthe, E. J. Baerends and J. G. Snijders, *The Journal of Chemical Physics*, 1993, **99**, 4597.
64. E. V. Lenthe and E. J. Baerends, *Journal of Computational Chemistry*, 2003, **24**, 1142-1156.
65. C. Fonseca Guerra, J. G. Snijders, G. te Velde and E. J. Baerends, *Theoretical Chemistry Accounts: Theory, Computation, and Modeling (Theoretica Chimica Acta)*, 1998, **99**, 391-403.
